# Organoid‐Guided Precision Medicine: From Bench to Bedside

**DOI:** 10.1002/mco2.70195

**Published:** 2025-05-01

**Authors:** Boqiang Tao, Xiaolan Li, Ming Hao, Tian Tian, Yuyang Li, Xiang Li, Chun Yang, Qirong Li, Qiang Feng, Hengzong Zhou, Yicheng Zhao, Dongxu Wang, Weiwei Liu

**Affiliations:** ^1^ Department of Oral and Maxillofacial Surgery Hospital of Stomatology Jilin University Changchun China; ^2^ Laboratory of Allergy and Precision Medicine Chengdu Institute of Respiratory Health the Third People's Hospital of Chengdu Affiliated Hospital of Southwest Jiaotong University Chengdu China; ^3^ Laboratory Animal Center College of Animal Science Jilin University Changchun China; ^4^ College of Basic Medicine Beihua University Jilin China; ^5^ Zhichuang Gene Editing Animal Model Research Center Wenzhou Institute of Technology Wenzhou China

**Keywords:** drug screening, organoids, precision medicine, regenerative medicine

## Abstract

Organoid technology, as an emerging field within biotechnology, has demonstrated transformative potential in advancing precision medicine. This review systematically outlines the translational trajectory of organoids from bench to bedside, emphasizing their construction methodologies, key regulatory factors, and multifaceted applications in personalized healthcare. By recapitulating physiological architectures and disease phenotypes through three‐dimensional culture systems, organoids leverage natural and synthetic scaffolds, stem cell sources, and spatiotemporal cytokine regulation to model tissue‐specific microenvironments. Diverse organoid types—including skin, intestinal, lung, and tumor organoids—have facilitated breakthroughs in modeling tissue development, drug efficacy and toxicity screening, disease pathogenesis studies, and patient‐tailored diagnostics. For instance, patient‐derived tumor organoids preserve tumor heterogeneity and genomic profiles, serving as predictive platforms for individualized chemotherapy responses. In precision medicine, organoid‐guided multiomics analyses identify actionable biomarkers and resistance mechanisms, while clustered regularly interspaced short palindromic repeats‐based functional screens optimize therapeutic targeting. Despite preclinical successes, challenges persist in standardization, vascularization, and ethical considerations. Future integration of artificial intelligence, microfluidics, and spatial transcriptomics will enhance organoid scalability, reproducibility, and clinical relevance. By bridging molecular insights with patient‐specific therapies, organoids are poised to revolutionize precision medicine, offering dynamic platforms for drug development, regenerative strategies, and individualized treatment paradigms.

## Graphical abstract

This review focuses on the structural and characterization aspects of organoids derived from original cells and scaffold materials. It also examines the construction methodologies, the incorporation of cytokines, and the practical applications in precision medicine. These applications include models for tissue development, drug screening and toxicity testing, disease modeling, personalized diagnostics, cell therapy, and regenerative medicine.

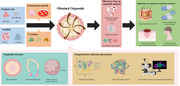

## 1 Introduction

Organoids, as an emerging biotechnology, have garnered significant attention in the field of precision medicine in recent years [1, 2]. This technology involves cultivating patient‐derived stem cells to create three‐dimensional (3D) miniature models that closely resemble actual organs [3, 4]. These organoids can effectively replicate the physiological characteristics and disease phenotypes of patients, thereby offering new possibilities for personalized treatment [5, 6]. The concept of organoid was proposed in 1907 [7]. Organoids are intricate 3D structures derived from somatic cells, adult stem cells (ASC)/progenitor cells, pluripotent stem cells (PSC), or specific cell lines that exhibit similar structures and functions to internal organs [8]. By simulating cell–cell and cell–matrix interactions, organoids can generate cell lineages resembling living tissues while maintaining genomic stability [9]. Organoids provide a more stable system for long‐term cultivation and manipulation of environmental factors, signaling pathways, and gene editing compared with in vivo animal models [10, 11, 12]. Although researchers have begun to investigate cellular behavior in 3D environments, the construction and application of organoids have been constrained by the absence of effective methods and tools. Since the onset of the 21st century, 3D cell culture technology has gradually matured [13, 14, 15]. 3D cell culture is a technique employed to cultivate cells or tissues in a 3D environment, aiming to mimic their physiological structure and environment in vivo [16]. In comparison with conventional two‐dimensional cultures, 3D culture provides an in vitro model that closely resembles the authentic tissue architecture and functionality [17]. The establishment of organoid culture heavily depends on the implementation of 3D culture technology [18]. To date, scientists have successfully utilized stem cell‐derived technology to generate various types of organoids, including normal tissue organoids (such as skin, brain, intestinal, and kidney organoids) as well as tumor organoids [19, 20, 21, 22]. These organoids not only provide new tools for basic research but also open up new avenues for clinical applications.

Precision medicine is a rapidly developing medical field focused on new treatment and prevention methods that are informed by an understanding of individual genes, environment, and lifestyle [23]. In contrast to traditional treatment methods, which often employ standardized protocols for all patients, precision medicine emphasizes the development of personalized treatment plans aimed at enhancing medical outcomes [24]. The swift advancement of precision medicine is largely attributed to the integration of genomics, the evolution of artificial intelligence (AI) and big data, and the creation of novel patient‐specific disease models [25, 26]. Among these innovations, organoids serve as in vitro models that can accurately replicate patient characteristics [27]. With the guidance of organoids, there is a greater likelihood that precision medicine will transition from the laboratory to clinical practice.

The advancement of organoid technology has elevated the field of precision medicine to new heights. Skin organoids, cultivated under the influence of cytokines, have become a pivotal tool in skin biology. These organoids are instrumental in models of skin development, providing valuable insights into the complex processes of skin differentiation and morphogenesis. While brain organoids have been utilized to investigate brain development, cardiac organoids have been employed to study heart development, an increasing number of organoids are being developed and utilized in precision medicine and personalized treatment [28, 29]. Organoids have significant applications in drug screening and toxicity testing. For instance, human colon organoids have been used to test the efficacy of flumazenil in mitigating radiation toxicity [30]. Similarly, skin organoids can be utilized to model Mpox virus infection and subsequent drug treatments, offering a human‐relevant system for preclinical testing of antiviral therapies [31]. In the field of cell therapy and regenerative medicine, the integration of skin organoids with animal skin components has proven feasible for the generation of transplantable skin constructs aimed at burn treatment and wound healing [19]. This approach represents a practical example of organoids in tissue engineering reconstruction, potentially leading to advancements in personalized medicine and regenerative therapies. In 2022, the US Congress approved the United States Food and Drug Administration (US FDA) Modernization Act 2.0, which eliminates the mandatory requirement for animal testing in preclinical drug development. This legislation promotes the use of alternative methods, including organoids, although the US FDA has not yet approved organoids for clinical use [32]. Despite this, the potential of organoids in clinical settings is garnering attention, and their eventual approval could herald a new era of personalized medicine and organ regeneration treatments.

Currently, research on organoids predominantly emphasizes basic science, with limited exploration transitioning from laboratory settings to clinical applications. This article centers on the application of organoids in clinical contexts, first summarizing the advancements in laboratory‐based organoid research. It then highlights organoid technologies that have either been implemented in clinical settings or possess potential for future application. Tumor organoids primarily contribute to precision medicine through drug screening, while normal tissue organoids hold broader possibilities in disease research, regenerative medicine, and other domains [33, 34]. Consequently, this review focuses on various types of organoids, supplemented by discussions on their applications in fields related to precision medicine, to illustrate the journey of organoids from laboratory research to preclinical exploration.

In this review, we will provide a comprehensive and systematic introduction to scientific research and preclinical studies involving organoids, while elucidating the connection between organoid‐guided research and precision medicine. We will begin by discussing the laboratory generation of organoid‐based models, covering aspects such as scaffolding, construction methods, original cell sources, cytokines, and the structural characteristics of the final organoid produced. Next, we will explore the laboratory techniques employed to detect organoids, describe the analysis of organoid data obtained through various methods, and examine the potential signaling pathways and cell–cell interactions involved by integrating bioinformatics and big data. Finally, we will summarize the practical applications of organoids in key areas of precision medicine, which include modeling tissue and organ development, drug screening and toxicity testing, disease modeling, personalized diagnostics, and cell therapy and regenerative medicine. Through this review, we aim to enhance the understanding of organoids within the context of precision medicine and clinical treatment, thereby facilitating the successful transition of various organoids from the laboratory to clinical practice.

## 2 Bench Breakthrough: Construction of Organoid Models

The technological innovation of organoids during the basic research stage in the laboratory serves as the foundation for precision medicine, which is driven by the clinical transformation of organoids. The construction of organoids should take into account multiple factors, including cell types, scaffolds, construction strategies, and endogenous and exogenous signals during the culture process. Variations in these factors will affect the outcome of organoid construction and impact their development and functionality. Simultaneously, diverse construction techniques will yield organoids or equivalents with varying structural morphologies.

### 2.1 Culture Scaffold

Scaffolding is a crucial component in the construction of organoids. In traditional two‐dimensional cell cultures, the cells usually proliferate as monolayers adhered to the base of petri dishes or culture bottles or exist individually within a culture medium [35]. Nevertheless, for enabling 3D cellular growth and faithfully reproducing the complex architecture of authentic tissues and organs, essential constituents encompass suitable scaffolds and microenvironments. Based on their source, frequently utilized matrix scaffold materials can be categorized into natural substances and synthetic counterparts.

#### 2.1.1 Natural Scaffold

The extracellular matrix (ECM) is a complex network structure composed of macromolecules, including collagen, enzymes, glycoproteins, and hydroxyapatite, which are secreted by cells [36]. It involves in facilitating cell adhesion and intercellular communication while providing the necessary environmental conditions for cellular activities and signal transduction. Additionally, the ECM actively participates in regulating diverse biological processes such as cell morphology, migration, differentiation, and regeneration [37]. Given the pivotal role of the ECM in organoids, researchers are actively exploring natural alternatives to ECM components [38]. Matrigel is a natural scaffold derived from Engelbreth‐Holm‐Swarm mouse sarcoma, which can effectively simulate the natural basement membrane and has been shown to promote the maturation of skin appendages [39]. Matrigel has found extensive application in the field of skin organoids [40], sweat gland organoids [41], and hair follicle organoids [42]. However, variations in the biochemical characteristics of different batches of Matrigel may lead to limited reproducibility [43]. Since Matrigel was derived from mouse sarcomas, there is a need to develop an ECM that can be used in humans for future clinical applications.

Apart from the utilization of Matrigel, decellularized ECM (dECM) produced by other human origin or animal origin cells have also been employed as an innovative natural material for organoid cultivation [44, 45, 46]. Animal‐derived dECM can be obtained from various tissues, including heart, liver, and lung [47, 48, 49]. Specifically, dECM derived from porcine ventricular tissue and fibrinogen has been utilized in a 3D cardiac cell culture model [50]. Furthermore, dECM sourced from porcine small intestinal submucosa has been employed in the culture of intestinal organoids [51]. Additionally, some human‐derived dECM is also utilized for organoid culture. For instance, human decellularized placenta‐derived dECM has been shown to promote the differentiation of spinal cord organoids into neurons and to accelerate their maturation process [52]. Moreover, dECM is widely used in the culture of skin organoids. The dECM scaffolds possess the capacity to repair and regenerate skin tissue by effectively retaining physical signals that facilitate keratinocyte adhesion and promote angiogenic cell proliferation [53, 54]. The ECM derived from dermal papilla cells undergo dynamic changes throughout the hair cycle in hair germ‐like organoids. Although these ECM alterations do not compromise the regenerative potential of human dermal papilla in inducing hair growth, they significantly influence cell proliferation [55]. Through dECM technology, the dental matrix of tooth bud tissue successfully forms enamel and dentin, and the acellular scaffold of glands can support the attachment of cells and the formation of glandular organs [56]. In general, ECM is widely used in organoid culture, which can simulate a variety of different special environments in vivo and contribute to the growth of organoid cells.

There are obvious defects in using natural material scaffolds to culture organoids. First of all, natural material scaffolds are often extracted from natural tissue, so the components extracted from different tissues are different [57]. Second, the composition of natural material scaffolds can only be used for scientific research, it is difficult to be used for clinical purposes. Finally, considering the inherent sources of natural material scaffolds and potential immunological challenges, it is imperative to develop natural material scaffolds that are suitable for clinical translation [58].

#### 2.1.2 Synthetic Scaffolds

Compared with natural scaffolds, synthetic scaffolds demonstrate superior mechanical stiffness and enhanced modifiability. Electrospun polymer fibers, specifically poly(l‐lactic acid), a biodegradable polymer known for its excellent biocompatibility, were electrospun into fibers exhibiting either smooth or porous topologies [59]. These distinct topographies are attributed to their inherent 3D structure, mimicking the ECM microenvironment and providing biochemical flexibility and mechanical support. Furthermore, the incorporation of 0.1% collagen onto both porous and smooth scaffolds significantly enhanced the adhesion and migration of keratinocytes and fibroblasts [60]. Sterile freeze‐dried collagen‐glycosaminoglycan scaffolds can serve as effective scaffold materials. Coculturing of sterile freeze‐dried collagen‐glycosaminoglycan scaffolds was conducted to generate engineered skin substitutes [61]. The scaffolds synthesized from the aforementioned these polymer materials are more effective in promoting cell adhesion for various cell types. Consequently, they are frequently utilized to create skin organoids composed of fully differentiated ASCs, such as keratinocytes and fibroblasts. The morphology of the skin organoid generated using these synthetic scaffolds is typically lamellar, indicating that the configuration of the skin organoid is influenced by the shape of the scaffold. Hydrogel, a type of biomaterial, holds significant potential in the construction of organoids. A hyaluronic acid–gelatin hydrogel for culturing patient‐derived organoids (PDO). By employing a 3D bioprinted spindle‐shaped collagen‐type I hydrogel supplemented with laminin 511 device to generate skin organoids, this culture system effectively mitigates organoid necrosis and results in a twofold enhancement of keratinocyte differentiation as well as an eightfold increase in hair follicle formation [62]. Hydrogel‐based synthetic scaffolds frequently serve as ECM substitutes, offering significant advantages in the construction of PSC‐derived skin organoids, which typically exhibit a spherical morphology. The utilization of synthetic scaffolds enables the circumvention of animal‐derived materials, thereby mitigating potential adverse effects.

#### 2.1.3 The Interaction Mechanisms Between Stem Cells and Scaffolds

EMC can contain several growth factors known to enhance the continuous cell proliferation and differentiation. The multiple fibrous proteins in EMC present a variety of integrin ligands that contribute to activation of signaling pathways that regulate the cell activity in organoids. Type I collagen in the ECM supports the maturation of human PSCs, while the laminin–entactin complex and fibroblast growth factors (FGF) 4 contribute to the generation of mature heart organoids from mouse embryonic stem cell (ESC)‐derived embryoid bodies (Ebs) [63, 64]. ECM scaffolds provide a cell‐instructive structural framework that mimics the structure and function of the ECM, delivering essential physical and chemical signals that guide cell differentiation and tissue formation. Evidence has shown that ECM can induce human PSCs (hPSCs) to express early markers associated with kidney development, such as *OSR1*, *PAX2*, *LHX1*, *HOXD11*, and *GATA3*, thereby promoting the formation and differentiation of kidney organoids [65]. Furthermore, the bioactivity and stiffness of the ECM significantly influence cell behavior and tissue formation. During the cultivation of ceramic organoids, it was observed that fibrous morphologies enhance forebrain identity. The differences in the ECM nanofibrous structures of various peptide amphiphiles will ultimately result in morphological variations in the Ebs induced by hPSCs, which will, in turn, influence the transition from Ebs to ceramic organoids [66].

### 2.2 Construction Method

**FIGURE 1:**
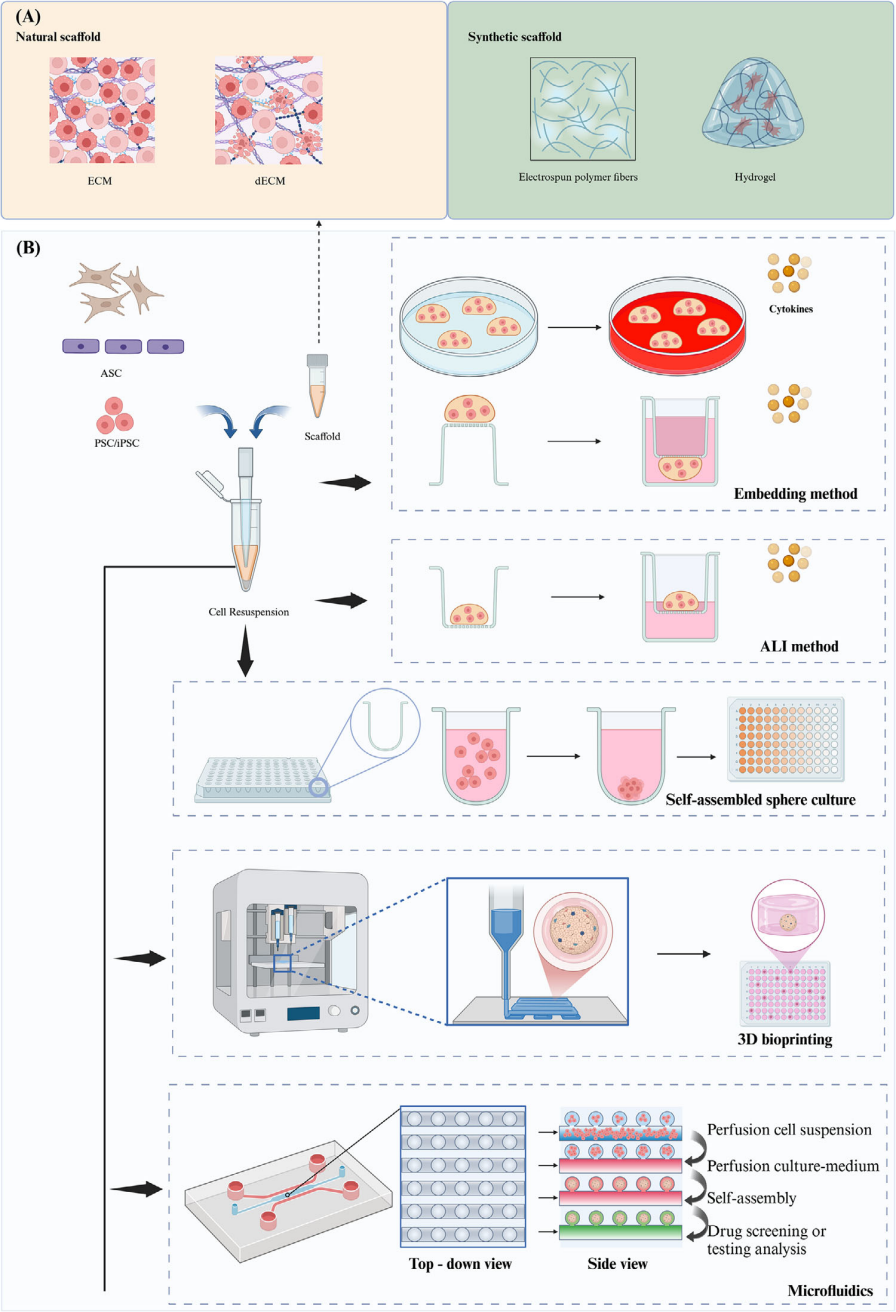
Generation of organoids. Different construction methods and scaffold materials of organoid. The dashed arrow indicates the types of scaffolds (A), which can be categorized into two major groups: natural scaffolds and synthetic scaffolds. Organoids are generated from PSC/iPSC or ASC as the original cells, and various construction methods incorporate different types of scaffolds (B). The blue arrow illustrates the combined addition of original cells and scaffolds. The thick black arrows, along with the dashed boxes they point to, represent different construction methods, including the embedding method, air–liquid interface (ALI) method, self‐assembled sphere culture, 3D bioprinting, and microfluidics. The thin black arrows and black curved arrow outline the steps in the construction process. Image created in BioRender.com.

There are a variety of methods to construct organ‐organoids or 3D culture, such as embedding in domes formed by matrix glue or ECM scaffolds and immersing them in specific cell culture media, or using low‐adsorption round‐bottomed petri dishes and bioreactors (Figure 1).

#### 2.2.1 Embedding Method

In the embedding method, the cells are completely surrounded by matrix glue and solidified to form a 3D shape, which makes the cells promote cell expansion, differentiation, and maturation in a microenvironment more similar to that in vivo. Specifically, the cell–Matrigel mixture was directly dispensed onto 24‐well or six‐well plates, or on the lower surface of Transwell plates with 0.4 µm pore polycarbonate membranes, and allowed to solidify into droplets at 37°C. Subsequently, these droplet structures were immersed in complete medium for continuous cultivation [67, 68]. However, because the matrix glue does not contain other endogenous and exogenous cytokines, it is often necessary to add additional growth factors or cytokines to the submerged medium. This technique has been employed for the 3D culture of various tumor organoids, sweat gland organoids [67], and skin organoids [40]. The advantages of this culture method are that the culture medium can be easily changed and single stem cell‐derived organoids can be obtained. For organoids that require long‐term continuous culture, it is easy to destroy the structure of organoids during passage.

#### 2.2.2 Air–Liquid Interface Method

The air–liquid interface (ALI) method is a widely employed technique in organoid culture, enabling the simulation of an in vivo environment through interactions between air and liquid. The ALI method necessitates the use of additional sleeves [69]. Initially, a gel containing similar organs or cells is introduced into the inner culture chamber to form a cell‐gel layer. Subsequently, the culture medium is added to the culture plate, ensuring that its horizontal level does not surpass the upper cell gel layer. This arrangement allows for exposure of cells to both liquid and ample gas [70]. For certain cells or organoids with high oxygen requirements, such as lung organoids [71], tumor organoids [72], and intestinal epithelial stem cells [73], the ALI culture methood is more suitable and provides sufficient oxygen. They can be induced to form stratified epidermal structures at the ALI in suitable in vitro culture medium for skin epidermal cells, and sweat gland‐like structures can also be generated [41]. The utilization of this technique enhances the oxygen supply to the organoids, enabling them to closely mimic the physiological conditions of the skin.

#### 2.2.3 3D Suspension Culture and Self‐Assembled Sphere Culture

The utilization of self‐assembling spheres as a culture technique enables the spontaneous formation of 3D multicellular spheres through suspension or rotary table culture. Suspension culture typically employs an ultra‐low sorption round bottom Petri dish with a specialized coating to maintain cells in an unattached state, thereby facilitating sphere formation via cell–cell junctions. This approach more accurately recapitulates cellular interactions and communication within tissues, leading to the generation of spherical organoids, such as colorectal cancer (CRC)–PDO and cerebral organoid [74, 75]. The drawback of this culture method lies in the potential reduction in diameter of organoid spheroids due to compaction facilitated by homotypic cell adhesion [76]. Due to its simplicity and controllability, as well as the ability to perform it in ultra‐low attachment 96‐well plates or larger quantities of plates, this method is also highly advantageous for procedures such as drug screening [74]. The method described herein offers a distinct advantage in the early acquisition of a precise quantity of spherical organoids, facilitating efficient drug screening.

#### 2.2.4 3D Bioprinting

3D bioprinting technology enables the design and precise printing of biological materials and cells into specific 3D tissue structures, thereby more accurately replicating the anatomical and functional characteristics of human tissues. Consequently, the 3D bioprinting of organoids has become a vital tool in regenerative medicine, drug research, and personalized medicine. Bioinks used for 3D bioprinting organoids consist of biocompatible materials and cell suspensions. By manipulating the hydrogel in 3D bioprinting, hydrogel constraints can enhance cell polarity in liver organoids, direct the morphogenesis of small intestinal organoids, and regulate lung tip bifurcation based on the hydrogel's composition and shape [77]. The application of 3D bioprinting for the production of organoids can significantly enhance large‐scale organoid generation, allowing for rapid and high‐throughput production. This technique facilitates the creation of organoids with consistent cell numbers and viability, making them well suited for drug screening purposes. Additionally, 3D bioprinting allows for precise manipulation of various biophysical properties, including organoid size, cell number, and conformation [22]. The 3D bioprinting of organoids integrates engineering technology with biology, offering a viable approach to address the complexities of tissue structure, precision, and large‐scale production. This innovative technique demonstrates significant potential in the fields of precision medicine, drug development, and regenerative medicine.

#### 2.2.5 Microfluidics

Microfluidics is a technology that precisely controls nanoliter to microliter volumes of fluid through a network of micrometer‐sized channels. The ability of microfluidic technology to manipulate microfluids with precision and its high‐throughput capabilities align well with the practical needs of organoid research. Consequently, the application of microfluidic technology in organoid research allows for the accurate control of physical and chemical parameters in the culture environment, including nutrient concentration, CO_2_ levels, and waste excretion [78]. Microfluidic multiphase flow droplet technology generates droplets by manipulating two or more immiscible liquids within microchannels, facilitating their merging or entrainment [79]. This technology is capable of producing rapid, ultra‐high‐throughput droplets, effectively reducing reagent consumption and ensuring the consistency of cultured organoids [80, 81]. An enhanced approach involves hydrogel organoid culture, which utilizes a hydrogel cell solution as the encapsulating aqueous phase. In this method, cells are presuspended in the hydrogel solution, and a microfluidic sandwich flow is employed to generate hydrogel droplets containing the cells. As the hydrogel solidifies, it forms solid‐phase cell gel spheres [82]. These cell gel spheres are then cultured in the aqueous phase to develop organoids. The aqueous solution surrounding the gel spheres continuously supplies the cells with essential nutrients while also removing cellular metabolic waste, enabling the long‐term culture of organoids [83, 84]. Another advantage of microfluidics in the study of organoids is its capability to investigate diseases affecting both organs and their potential treatments. Using a microfluidic system, cardiac microtissues and kidney organoids derived from human induced PSCs (iPSCs) were generated and loaded into two separate, communicating chambers of a perfusion chip. This system allows for the in vitro study of the interactions between human heart and kidney organoids while controlling parameters such as fluid flow speed and direction [85]. Microfluidic technology offers a highly efficient, precise, and controllable approach for the generation of organoids, thereby advancing the fields of precision medicine and regenerative medicine.

### 2.3 Original Cell

The majority of organoids are derived from PSCs and ASCs, which serve as valuable original cell materials due to their capacity for pluripotent differentiation and self‐renewal. Stem cells possess the ability to undergo self‐organization and differentiation within a 3D environment, giving rise to functional structures resembling authentic biological tissues [86]. The two types of stem cells, ASCs and PSCs, hold significant importance in the field [87, 88]. While ASCs demonstrate limited differentiation potential confined to specific tissue types, PSCs exhibit robust differentiation capabilities that enable them to differentiate into any human cell type [89].

ASCs, also referred to as tissue stem cells, are undifferentiated cells derived from specific tissues. Their origin and capacity for self‐renewal enable them to undergo differentiation into cell types that are specific to the respective tissues [90]. Tissue‐specific ASCs possess an inherent ability to spontaneously generate corresponding organs, thereby reducing uncertainties associated with cellular differentiation in a microenvironment and mimicking the development of actual organs in vivo [91]. Consequently, ASC‐derived organoids can effectively replicate steady‐state or regenerative conditions of their original tissues, making them valuable tools for studying tissue biology [92]. For instance, dental pulp stem cells sourced from dental pulp tissue exhibit excellent odontogenic potential and can be utilized for generating tooth germ organoids [93].

PSCs possess an extraordinary capacity for pluripotent differentiation and self‐renewal, enabling them to differentiate into a wide range of cell lineages. PSCs can be derived from iPSCs or obtained from early embryos, and they are extensively utilized in the construction of organ‐organoids across various tissues [94]. However, the precise control of embryonic development and the complex differentiation conditions in analytical development are still challenging to achieve in vitro. Further research is imperative to elucidate optimal conditions for their directed differentiation and development before widespread application of PSC‐derived organoids can be realized [95]. Nevertheless, under appropriate medium and scaffold conditions, specific tissue‐specific organoids such as human cortical [96], liver [97], and skin can currently be generated using PSCs [19]. Given that PSC‐derived organoids faithfully recapitulate key aspects of embryonic developmental processes, they offer an unparalleled advantage in investigating intricate cell–cell interactions, mechanisms of organogenesis, and developmental defects [98]. Through the addition of a variety of cytokines, PSC differentiate into salivary gland progenitors, which can eventually form PSC‐derived salivary glands organoids [99].

Compared with PSC‐derived organoids, ASC‐derived organoids can be obtained more easily and cultured in vitro for a shorter duration [87]. Although the differentiation process of PSC‐organoids is relatively intricate, requiring specific signaling molecules for induction and an extended culture period, PSCs possess the advantage of differentiating into diverse cell lineages that enable them to produce various types of tissues. This ability overcomes the limitations associated with ASCs [100].

### 2.4 Cytokines

**FIGURE 2:**
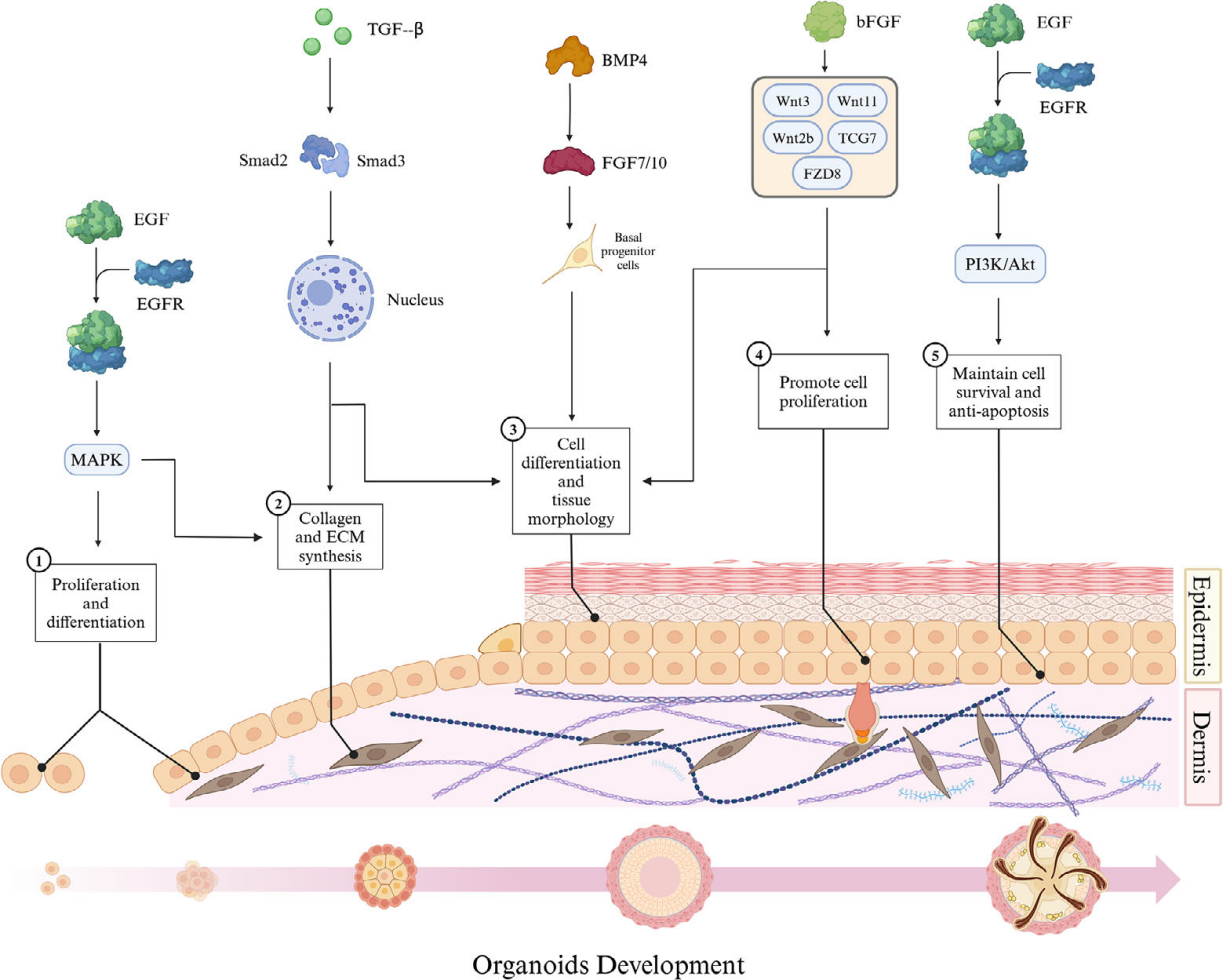
Cytokines influence the development of organoids. Different types of cytokines play a variety of roles in organoid culture. Below is a schematic of the organoid structure over time. At the top are the pathways of action of different cytokines. Cytokines include EGF, TGF‐β, BMP4, bFGF, and so on. The pink arrow below illustrates a schematic diagram depicting the structural changes of organoids over time. The lines with black dots indicate the locations where biological processes occur, while the black arrows represent the actions of cytokines. Image created in BioRender.com.

The formation of organoid is usually regulated by endogenous and exogenous signals, including growth factors and molecules. The formation of skin organ culture model in vitro involves a number of signal regulation processes. The latest research has demonstrated the crucial involvement of transforming growth factor‐beta (TGF‐β), bone morphogenetic protein 4 (BMP4), and FGFs signaling pathways in the in vitro differentiation of organoids (Figure 2).

#### 2.4.1 Transforming Growth Factor‐beta

TGF‐β is a multifunctional signaling molecule that exerts regulatory effects on various cell types and biological processes such as cell–cell interactions in corneal endothelial cells and the formation of organ‐like structures in mammary epithelial cells [101, 102]. This phenomenon also applies to the occurrence of skin organs [103]. The action of TGF‐β is mediated through Smad‐dependent signaling pathway. Upon binding to its cell surface receptor, TGF‐β phosphorylates the Smad proteins, namely, Smad2 and Smad3, facilitating their translocation into the nucleus where they regulate the synthesis and secretion of ECM components, including collagen type I [104]. In the process of skin development and repair, TGF‐β regulates the proliferation, differentiation, and migration of epidermal and dermal cells [105, 106]. It promotes the formation and maturation of the embryonic epidermal layer, as well as the expansion of basal cells. Furthermore, it stimulates the proliferation and differentiation of later epidermal cells and hair follicle stem cells [19]. Additionally, TGF‐β modulates dermal cell function and ECM synthesis by promoting collagen and elastin production and accumulation, thereby providing structural support [107]. Moreover, TGF‐β also governs inflammatory processes, immune responses, skin injury repair mechanisms, as well as scar formation [108]. While TGF‐β is known to play a significant role in the formation and development of hair follicles within skin organoids, the premature introduction of TGF‐β signaling can negatively impact both the quantity and size of early skin organoids. Inhibiting TGF‐β can prevent premature or inappropriate cell differentiation, thereby aiding in the preservation of organoid pluripotency and plasticity [109]. In cultures of lung organoids, the activation of TGF‐β impaired the ability of fibroblasts to support the formation of adult lung epithelial progenitor cell organoids by inducing myofibroblast differentiation and altering the expression of key components in signaling pathways [110]. Genome‐scale clustered regularly interspaced short palindromic repeats (CRISPR) screening of intestinal organoids confirmed that the TGF‐β pathway regulates the growth of these organoids and identified key drivers of TGF‐β resistance [111]. Overall, these studies highlight the significance of TGF‐β in organoid culture systems and its role in various biological processes, including cell differentiation, ECM synthesis, and disease pathogenesis.

#### 2.4.2 Bone Morphogenetic Protein 4

BMP4 is implicated in the regulation of cell lineage differentiation and morphogenesis and has been utilized to facilitate organoid formation [112]. BMP4 accelerates the growth of basal progenitor cells by activating the downstream FGF7/FGF10 pathways and directs epidermal fate in the presence of WNT pathways [113]. First, BMP4 is involved in regulating embryonic skin development by promoting proliferation and differentiation of epithelial cells during the embryonic period, thereby facilitating mature epidermis formation [114]. Second, BMP4 exerts concentration‐dependent regulation on cell development and morphology, thereby facilitating the generation of distinct epithelial organs, such as epidermis (surface ectoderm) or neuroepithelium [115, 116, 117]. During the initiation phase of hair follicle formation, BMP4 orchestrates the differentiation of epithelial cells into specialized hair follicle cells while concurrently supporting the proliferation and maintenance of hair follicle stem cells [114]. Furthermore, BMP4 governs dermal cell differentiation and fosters dermal tissue formation [118]. Similar to TGF‐β, BMP‐related proteins typically require inhibition during the early stages of skin organoid culture. The antagonist Noggin is commonly employed to bind and inhibit the activity of BMPs, thereby establishing a microenvironment that supports stem cell self‐renewal in initial organoid cultures [19]. In the context of inner ear organoid culture, BMP4 has been shown to promote non‐neural induction from mouse PSC [119]. In lung alveolar stem cells, niche‐mediated BMP/SMAD signaling has been shown to regulate the support function of alveolar type (AT) 2 cells in organoids, both after partial pneumonectomy and in culture [120]. Additionally, the functional development of mechanosensitive hair cells in stem cell‐derived organoids has been demonstrated to parallel native vestibular hair cells, indicating the potential for generating fully functional cells in a 3D culture system [121]. Furthermore, the addition of Chk1 inhibitor and BMP4 has been found to cooperatively promote the development of retinal organoids with encapsulated neural retina tissue in retinal pigment epithelium in long‐term culture [122]. In kidney organoids, asymmetric BMP4 signaling has been shown to improve the realism of nephrons, although the absence of a ureter remains a significant unrealistic feature [123]. Moreover, BMP4 gradient along the intestinal villus axis has been found to control zonated functions in intestinal organoids, highlighting the importance of BMP signaling in these cultures for functional studies [124]. In liver organoid culture, BMP4 has been identified as using different effector pathways to regulate various functions [125]. Overall, BMP4 plays a crucial role in the development and function of organoids in various organ systems, influencing cell differentiation, tissue morphology, and physiological activity in culture.

#### 2.4.3 Fibroblast Growth Factors

FGFs are a group of multifunctional cell signaling molecules that activate intracellular signal transduction pathways by binding to FGF receptors (FGFRs) and play a crucial role in regulating the generation of skin [126]. The extracellular signal‐regulated kinase/mitogen‐activated protein kinase (MAPK) signaling cascade is activated by FGFs, stimulating epidermal stem cell proliferation in vitro [127, 128, 129]. Consequently, during the first week of skin organoid culture, the absence of FGF1 prevents organoid formation [109]. However, FGF10 can substitute for FGF1 by activating FGF receptor 1b, making the addition of FGF10 to the culture a viable alternative [109]. Basic FGF (bFGF) also acts in the formation of skin organoids, acting as a crucial cytokine involved in regulating cell proliferation and differentiation. In fibroblasts, several typical Wnt pathways genes, including Wnt2b, Wnt3, Wnt11, t cell factor 7 (TCF7), and crimp 8 (FZD8), under the stimulus bFGF expression level are changed [130]. During the process of skin organogenesis, bFGF exerts its effects through diverse role. First, it facilitates epithelial cell proliferation and guides their differentiation into mature epidermal cells, thereby contributing to the development of a functional epidermis [109]. Second, bFGF actively participates in hair follicle morphogenesis by maintaining hair follicle stem cell function and promoting self‐renewal and regeneration capabilities [19]. Additionally, bFGF is implicated in dermal formation by stimulating dermal cell proliferation and differentiation while enhancing collagen synthesis and elastic fiber production for establishing a healthy dermis [131]. Overall, bFGF orchestrates various processes including epithelial cell dynamics, hair follicle development, and stem cell maintenance to promote skin organogenesis.

Furthermore, research by Otte et al. [132] investigated the role of FGF2 in the self‐renewal of highly malignant CRC organoids. The study found that FGFR‐inhibition affected organoid culture, emphasizing the significance of FGF signaling in maintaining organoid function [132]. In addition to intestinal organoids, FGFs have also been shown to play a role in hepatic differentiation in liver organoid culture [125]. The proproliferative effect of FGFs, including FGF‐10, has been demonstrated in 3D cell culture models, where FGF signaling enhanced lung organoid formation [133]. Overall, FGFs, particularly FGF‐2 and FGF‐10, have been identified as crucial factors in maintaining the self‐renewal capacity and cellular diversity of various organoids, including intestinal and CRC organoids. Further research is needed to fully understand the specific mechanisms by which FGF signaling influences organoid culture and function [134].

#### 2.4.4 Epidermal Growth Factor

Epidermal growth factor (EGF) also participate in the process of skin organogenesis. Similar to FGF, EGF plays a crucial role during the initial week of skin organoid culture [109]. First, EGF functions as a signal transducer during early skin development. Upon binding to its receptor, it initiates a cascade of signaling pathways, including MAPK and phosphatidylinositol‐3 kinase (PI3K)/protein kinase B (Akt), which promote cell proliferation and differentiation, thereby facilitating the formation of skin [135]. Second, EGF significantly influences hair follicle development by stimulating stromal cell proliferation and epidermal cell differentiation, ultimately contributing to hair follicle formation [136]. Additionally, EGF modulates the self‐renewal and differentiation of hair follicle stem cells while maintaining their functionality [136]. Last, EGF impacts dermis formation by promoting dermal cell proliferation and collagen synthesis, thus supporting the generation of healthy dermal tissue [137].

In intestinal organoids, EGF has been known for its role in promoting the proliferation of intestinal epithelial cells [138]. Similarly, in salivary gland organoid culture, EGF has been shown to maintain distinct glandular secretory function during long‐term culture [139]. Furthermore, studies have highlighted the importance of growth factor quality in organoid cultures, with EGF being one of the essential factors for differentiation and maintenance of organoids [140]. The combination of EGF with other factors such as insulin‐like growth facto has been shown to enhance the recovery and self‐renewal capacity of organoids [134]. In addition to its role in promoting proliferation and self‐renewal, EGF has also been implicated in driving organoid growth through Wnt and Neuregulin1/ErbB signaling pathways [141]. This highlights the complex interplay between EGF and other signaling pathways in regulating the growth and function of organoids. Overall, the literature suggests that EGF plays a critical role in the culture of organoids across different tissues and organ systems, influencing their proliferation, differentiation, and maintenance of function. Further research is needed to fully understand the mechanisms underlying the function of EGF in organoid culture and its potential therapeutic applications [142].

### 2.5 The Main Types and Structural Characteristics of Organoids

**FIGURE 3:**
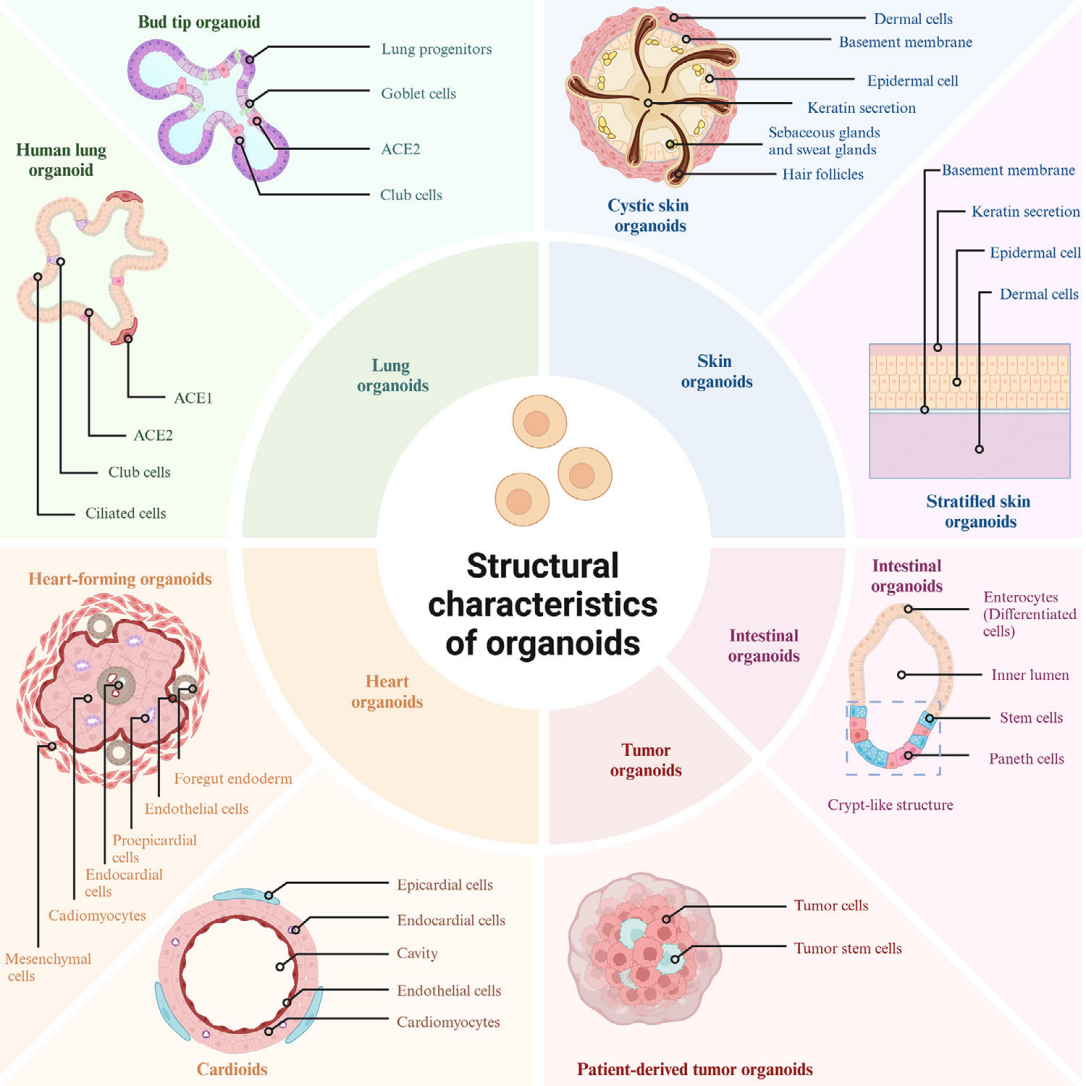
Schematic diagram of the structure of organoids. The structure of different types of organoids were demonstrated. The primary types of organoids presented include skin organoids, intestinal organoids, tumor organoids, heart organoids, and lung organoids. The inner ring represents the types of organoids, while the outer ring illustrates the corresponding organoids at various degrees of differentiation. The circle at the end of the line indicates where the structure or cell is located. Image created in BioRender.com.

Various types of organoids have been developed to model different tissues and diseases, each with unique characteristics and applications. Currently, through diverse research efforts, scientists have significantly enhanced various organoid construction methods. By selecting suitable scaffolds and stem cells, administering the appropriate cytokines at the optimal times, and employing effective construction techniques, it is now possible to create desired organoids in the laboratory, whether in spherical organoid, or other specific shapes. Given the diverse types of organoids, each characterized by distinct cellular components and structures, this review has selected a variety of organoids for introduction (Figure 3).

#### 2.5.1 Skin Organoid

One primary type of organoid is the skin organoid, which has been utilized in dermatological research to investigate various aspects of skin development and diseases [143]. The structure of skin organoids closely resembles that of the skin, comprising the epidermis and dermis, along with components such as the stratum corneum, hair follicles, sebaceous glands, and sweat glands [144]. The epidermis consists of a stratified epithelium composed of two main cell types: the basal layer containing epidermal stem cells and the superficial layer comprised of specialized keratinocytes responsible for keratin production. Epidermal stem cells continuously regenerate the epidermis through proliferation, which occurs every 40–56 days [145]. As these proliferating cells migrate outward, they undergo differentiation to form distinct layers within the epidermis including the stratum spinosum, granulosum, and corneum [146]. Located beneath the epidermis is a thick connective tissue layer called the dermis that contains collagen fibers, elastic fibers, and blood vessels. It lies below the basement membrane of the epidermis and provides support for both the epidermis and its appendages [147].

The morphologies of skin organoids encompass a spherical shape as well as a stratified structure skin to that of normal skin. Skin organoids can be generated by coculturing dermal and epidermal cells in specific ratios [148]. Skin organoids can also be generated by culturing PSCs or iPSCs in a matrix such as Matrigel [19]. Initially, these organoids adopt a cystoid morphology with three to four layers of dermal cells forming the outermost layer. Basement membrane formation is observed between the epidermal and dermal cell layers. The inner layer consists of epidermal cells while keratin secretion primarily occurs in basal rather than suprabasal epidermal cells within the central region of these spherical structures. Over time in culture, fusion among these organoids leads to the development of bilaterally symmetrical double‐layered epidermal planes. Subsequently, hair plate‐like structures are induced within the dermis [19, 148].

In addition to cystic skin organoids, the generation of stratified skin organoids with structures resembling normal skin has become feasible. The ALI method is currently the predominant technique for in vitro cultivation of stratified skin organoids. By employing type I collagen or other scaffold materials on a supportive substrate, distinct layers of keratinocytes and fibroblasts can be cultured, resulting in the formation of a stratified skin structure [149, 150]. Furthermore, the utilization of animal‐derived dECM facilitates the construction of visibly layered structures with human epidermal keratinocytes seeded atop and dermal structures containing human dermal fibroblasts at the base [151]. Planarization of human skin can also be achieved in vivo through transplantation of cystic form skin organoids into mice, wherein planar skin structures are observed to develop [40].

#### 2.5.2 Intestinal Organoids

Intestinal organoids accurately replicate the cellular composition and structural architecture of native intestinal epithelium. This fidelity enables the study of intestinal biology, disease mechanisms, and treatment responses in a controlled in vitro environment. Intestinal organoids are typically isolated from intestinal stem cells or intestinal crypt cells and subsequently cultured in vitro. Intestinal epithelial tissue consists of crypts and villi [152]. During intestinal development, the base region of the villi extends into the mesenchymal tissue, forming crypts. The bottom of the crypt, referred to as the stem cell zone, contains both stem cells and Paneth cells [153]. Intestinal organoids can be obtained by isolating crypt structures and conducting 3D culture. These organoids take the form of 3D microtissue spheroids, characterized by an inner lumen that faces inward and an outer surface that contacts the ECM, which surrounds the lumen. In the basal region of the intestinal organoid, a crypt‐like structure houses stem cells and Paneth cells, where the stem cells continue to proliferate and differentiate into various intestinal cell types, thereby maintaining the self‐renewal of the organoid and ensuring tissue homeostasis. A bulged state is typically reached around day 3.5 of 3D culture, transitioning to a budding state around day 4, during which part of the epithelium protrudes outward to form crypt buds [21].

#### 2.5.3 Lung Organoids

Lung organoids have emerged as a valuable system for investigating lung development, disease mechanisms, drug responses, and regenerative therapies. These organoids closely replicate the cellular diversity and microenvironment of the lung in vivo, thereby offering a physiologically relevant model for research. Lung organoids can be differentiated from 3D ventral–anterior foregut spheroids generated from human PSCs. When ventral–anterior foregut spheroids are cultured in high levels of FGF10 and 1% fetal bovine serum, they differentiate into airway‐like epithelium, which is surrounded by mesenchymal and epithelial cells that express markers characteristic of alveolar cell types, ultimately resulting in the production of human lung organoids. In contrast, when ventral–anterior foregut spheroids are cultured in a serum‐free environment containing FGF7, CHIR‐99021, and all‐trans retinoic acid, they yield bud tip progenitor organoids [154]. Lung organoids have the capability to recreate both bronchial and alveolar structures. Airway‐like structures mimic the tubular formations of bronchial epithelium, typically characterized by a pseudolamellar arrangement of ciliated and secretory cells. In contrast, alveolar‐like structures resemble spherical, sac‐like areas of alveoli, featuring distinct populations of AT1 and AT2 cells [155]. Lung organoids typically develop a hollow lumen that is surrounded by organized epithelial cells. This lumen facilitates the modeling of air–fluid interfaces that resemble those found on airway surfaces. Epithelial cells within lung organoids preserve apical–basal polarity, exhibiting functional localization of surface proteins that are essential for barrier function and effective signaling [156].

#### 2.5.4 Heart Organoids

Heart organoids replicate the cellular composition and tissue architecture of the human heart at various stages of development, making them essential models for simulating cardiac development and examining drug toxicity. Currently, miniature hearts have been constructed using human PSCs. By utilizing biological or chemical signals embedded in the hydrogel matrix, the differentiation of hPSCs can be precisely controlled, enabling 3D cell aggregates to develop into three‐layer, cup‐shaped structures known as heart‐forming organoids (HFO) within a span of 10–14 days. This three‐layer structure is not merely a random collection of cardiomyocyte clusters; rather, it is a complex assembly comprising at least seven distinct and well‐defined cell and tissue types [157]. Similar to the developing heart in vivo, heart organoids comprise a diverse array of key heart‐related cell types, including cardiomyocytes, endothelial cells, cardiac mesenchymal cells, and proepicardial cells [157]. Heart organoids typically manifest as spherical or layered structures containing lumens. HFO will initially present with numerous small cavities, which subsequently merge to form larger cavities. The cavity in the middle layer of HFO resembles the cavity formation of the mesoderm during human heart development, while the cavity in the inner layer at day 10 parallels the foregut lumen and vascular lumen. Certain models exhibit layered architectures that resemble the endocardium, myometrium, and media, thereby reflecting the stratification of the early heart [157]. Concurrently, cardiomyocytes within heart organoids are interconnected, enabling synchronous beating through electrophysiological signals and muscle contractions, which parallels the functionality of natural cardiac tissue. Some advanced heart organoids are capable of partially reconstructing the hierarchical architecture of embryonic ventricles and atrial precursors, demonstrating specific regional divisions of labor, such as the initial differentiation of the epicardium, endocardium, and myocardium. Under optimized culture conditions, heart organoids can develop capillary‐like microvascular structures, effectively simulating the heart's vascular network. Current heart organoids still exhibit limitations, including the inability to fully replicate the complex anatomical structures of the human heart, such as complete atria, ventricles, and valves, as well as insufficient vascularization and functional maturity.

#### 2.5.5 Tumor Organoids

As 3D culture technology continues to advance, researchers are increasingly applying this technology to cancer research by utilizing cells from various sources, primarily patient‐derived tumor cells, to establish diverse types of tumor organoid models. These patient‐derived tumor organoid (PDTO) models not only thrive in vitro but also preserve the genetic characteristics and microenvironment of the tumor, making them a crucial tool for investigating cancer development and progression. Given the similarities in the construction of PDTOs, they will not be described separately. Cancer organoids currently under investigation typically refer to PDTOs. These organoids are derived from primary patient material. Following processing, which includes chopping and cell digestion, a cell suspension is created and inoculated into scaffold materials, such as basement membrane extract, for 3D culture [158]. The resulting PDTO model primarily consists of the corresponding tumor cells and cancer stem cells, often organized into spherical or cystic structures [159, 160]. To more accurately simulate the tumor microenvironment, tumor‐associated fibroblasts and immune cells may also be incorporated during the coculture process of PDTOs [160, 161].

In conclusion, organoids represent a versatile and powerful tool for studying various tissues and diseases. From trophoblast organoids modeling maternal–fetal interactions to brain organoids mimicking lysosomal storage disorders, different types of organoids offer unique insights into organ development, disease pathology, and therapeutic interventions. By utilizing organoid models, researchers can advance our understanding of complex biological processes and develop novel strategies for disease treatment and prevention.

## 3 Bridging Bench and Bedside: Technical and Theoretical Basis

The advancement of precision medicine, informed by organoids, is inherently linked to the technical and theoretical foundations associated with organoids. The technical and theoretical aspects of organoids have effectively bridged the gap between laboratory research and clinical needs. This includes the collection and analysis of organoid data, the discovery and validation of biomarkers, and the theoretical principles grounded in cell–cell interactions and molecular mechanisms.

### 3.1 Methods of Organoid Data Collection and Analysis

**FIGURE 4:**
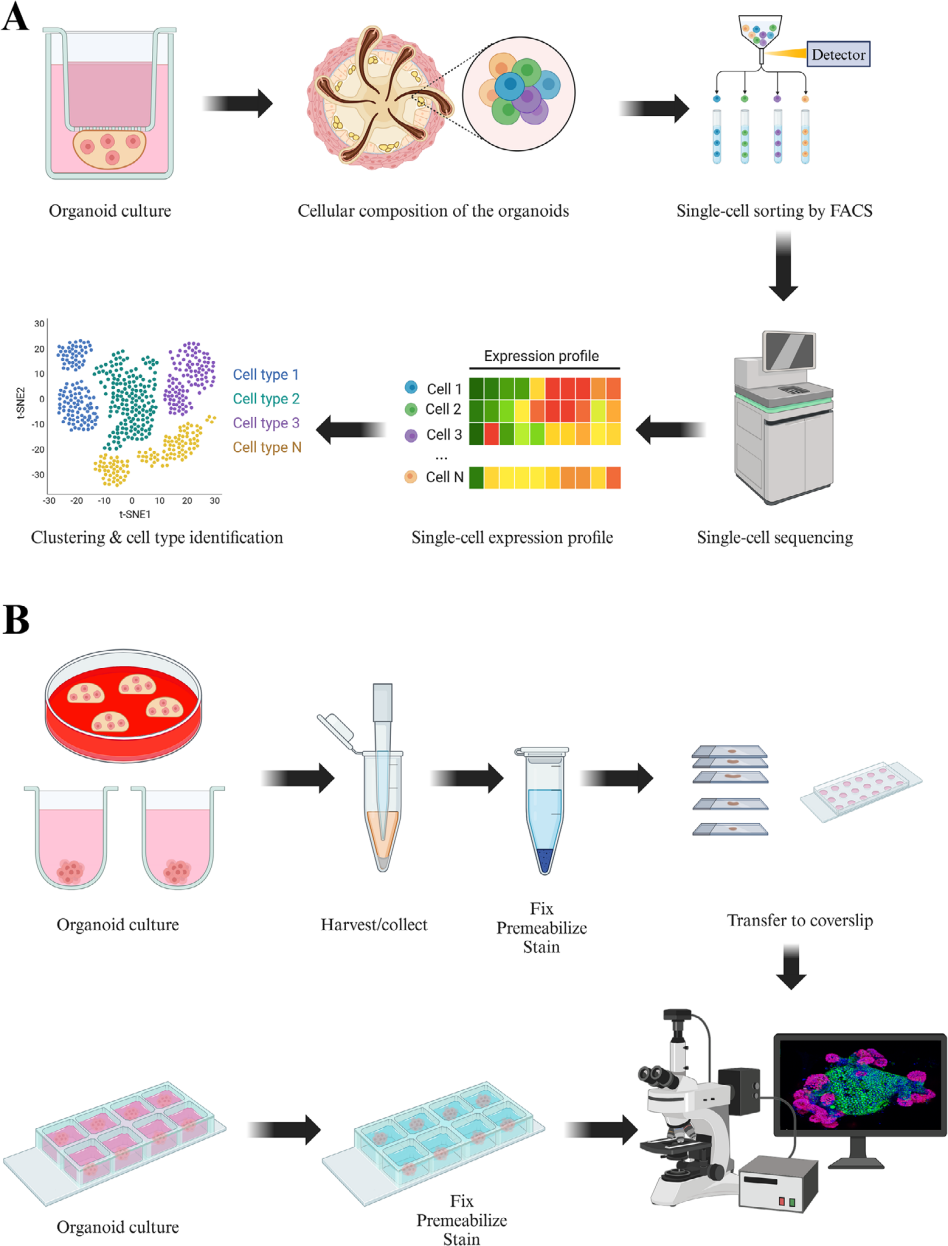
Characterization of organoid. Different characterization methods of organoids. (A) Application process of single‐cell sequencing in organoids. (B) Application process of immunofluorescence in organoids. The black arrows represent the sequence of processes. Image created in BioRender.com.

Due to the limited volume of laboratory‐constructed organoids, accurately evaluating their characteristics using the naked eye alone is challenging, necessitating the support of advanced laboratory technologies. Precision medicine guided by organoids relies on a combination of contemporary biotechnological and medical advancements, including cell staining techniques, high‐throughput screening methods, as well as genomics and data analysis support [162, 163, 164, 165]. Single‐cell sequencing offers distinct advantages in differentiating various cell subpopulations and acquiring spatial information regarding gene expression [166, 167]. Cell staining techniques such as immunofluorescence and immunohistochemistry have emerged as straightforward and convenient options for organoid detection, allowing for direct observation and quantitative analysis [168, 169]. Consequently, this paragraph emphasizes single‐cell sequencing, genomics technology, immunofluorescence, and immunohistochemistry as the principal technologies (Figure 4).

#### 3.1.1 Single‐Cell RNA Sequencing

Traditional second‐generation sequencing (NGS) measures the average expression of a gene in all cell populations in cell culture, tissue, or organ samples, while single‐cell sequencing measures the genome of a single cell in a cell population [170, 171]. Through single‐cell sequencing, we can obtain the gene expression patterns of individual cells to identify different subpopulations of cells and cell types [172, 173, 174]. In the research of organoids, single‐cell sequencing is extensively utilized to elucidate the cellular composition, differentiation potential, growth mechanisms, and complexity of disease models. This technique is particularly valuable due to its capacity to analyze cellular heterogeneity and dynamic processes.

Single‐cell RNA sequencing (scRNA‐seq) is a powerful tool for the identification of multiple cell types and can accurately distinguish between different cell types present in organoids. scRNA‐seq can be used to construct cellular composition maps of organoids, including the identification of multiple cell types and the identification of cell subpopulation‐specific characteristics. Using scRNA‐seq allows precise identification of different cell types in organoids [175]. During the establishment of organoids, cells may have different differentiation states. Through scRNA‐seq, intermediate state cells in the differentiation lineage can be identified to construct a characteristic map of cell differentiation [176]. ScRNA‐seq can reveal differences between subpopulations within the same cell type. For example, cancer stem cells in tumor organoids have different expression characteristics from rapidly proliferating tumor cells, and their phenotypic characteristics and corresponding key genes can be analyzed through single‐cell sequencing [176, 177]. In intestinal organoids, researchers used scRNA‐seq to completely describe the specific gene expression profiles of stem cells, proliferating cells, and differentiated cells, and constructed a cell atlas of organoid development [178].

Single‐cell sequencing can be used to study the dynamics of organoid development or differentiation. Organoids can simulate the tissue or organ development process in the body, and single‐cell sequencing can record the dynamic changes of cell populations during this process in real time. In terms of time series analysis, single‐cell sequencing combined with developmental time series analysis can reveal the differentiation trajectory from stem cells to mature cells [179]. Pseudotime analysis can also be used to reconstruct cell differentiation lineages and identify transcription factors and signaling pathways in key differentiation pathways. Single‐cell sequencing can also be used to analyze signaling pathway regulation. In organoid culture, adding specific factors can guide cells to differentiate into a specific cell type. Single‐cell sequencing can track how different signaling pathways drive differentiation and proliferation processes to optimize culture conditions [180]. The investigation of skin organoids can benefit from the utilization of single‐cell sequencing technology. By employing scRNA‐seq on skin organoids, the researchers are able to evaluate their resemblance to developing human embryonic skin tissue, cross‐reference them with the human Cell Atlas database, and analyze interactions between epidermal and dermal cells [148]. Single‐cell sequencing identified alterations in cell movement, cell migration, and cell adhesion signaling pathways during the formation of skin organoids to unveil the gene expression characteristics of craniofacial skin development [19, 148].

Single‐cell sequencing can also be used to study cellular heterogeneity in organoids. Organoids often preserve tissue or tumor heterogeneity, and single‐cell sequencing provides powerful analytical capabilities to reveal this heterogeneity. Single‐cell sequencing analysis reveals gene expression profiles that are unique to distinct cell populations within skin organoids and normal skin tissue, thereby providing insights into their cellular heterogeneity and functional diversity [19, 181]. By comparing single‐cell transcriptomics data from in vitro skin organoids and in vivo human skin, we can elucidate the characteristics of skin organoids and their differences from normal skin. Many of the major cellular states present in vivo are also observed in skin organoids in vitro. Characterization of the basal (BAS) stem cell community reveals that both in vitro skin organoids and in vivo human skin are enriched in known BAS keratinocyte marker genes, including PTTG1, RRM2, keratin (KRT) 15, and proliferating cell nuclear antigen (PCNA). The proportions of BAS‐I and BAS‐II circulating cells are essentially similar between in vivo tissues and in vitro skin organoids. However, the number of cells in the BAS‐III cluster of in vitro skin organoids is more than 3.5 times greater than that in in vivo tissues. In contrast, the proportions of spiny (SPN) cell clusters SPN‐2, SPN‐4, and SPN‐5, as well as the granular (GRN) cell cluster GRN‐2, are lower in vitro skin organoids compared with the in vivo human skin state [181]. PDTOs retain the genetic and phenotypic characteristics of a patient's tumor. ScRNA‐seq allows for the examination of the heterogeneity among various tumor cell subpopulations within these organoids. In the context of pancreatic cancer organoids, single‐cell sequencing has revealed that the density of cancer‐associated fibroblasts is correlated with heightened inflammation in epithelial cells and the occurrence of epithelial‐to‐mesenchymal transition [182].

The application of single‐cell sequencing technology in organoid research serves as a powerful tool for analyzing cellular heterogeneity, differentiation dynamics, disease mechanisms, and drug action mechanisms within organoids. In the future, integrating multiomics data along with spatial omics will enhance the role of single‐cell sequencing in more complex organoid applications, offering valuable insights into the function of organoids in disease modeling. This advancement will establish a solid foundation for further developments in mechanism research and precision medicine.

#### 3.1.2 Spatial Transcriptomics

Although single‐cell sequencing offers high‐resolution information at the cellular level, its characteristic of having a “detached spatial location” results in the loss of direct analysis of tissue structure [183]. Consequently, the integration of single‐cell sequencing with spatial transcriptomics (ST) has emerged as a prominent trend in recent years.

Organoids are typically composed of multiple cell populations that create microenvironments with tissue‐specific and spatial distributions. ST technology allows for the simultaneous marking of gene expression in a spatial context and the analysis of cellular maps within complex organoids. By employing ST technology, researchers can elucidate the distribution of gene expression across various cell types and examine their positional relationships within organoids [184]. ST technology can establish spatial partition maps for complex or highly heterogeneous organoids, facilitating the investigation of how cells in each region collaborate. This includes examining the distribution of neurons and glial cells simulated in different brain regions within brain organoids [185].

ST offers robust technical support for the investigation of organoids characterized by complex 3D structures. This approach reveals not only the cellular composition and developmental processes of organoids but also their responses to drugs and the communication networks that operate both extracellularly and intracellularly from a spatial perspective. By integrating single‐cell transcriptional data with spatial omics data, researchers can effectively reconstruct the spatial distribution of cells within organoids and elucidate how these cells are organized into specific functional regions.

#### 3.1.3 Immunofluorescence and Immunohistochemistry

Immunohistochemistry and immunofluorescence techniques are invaluable for the detection and localization of specific protein molecules within cells or tissues [186, 187, 188].

In skin organoids where multiple cellular components coexist, immunohistochemistry and immunofluorescence can effectively differentiate between these components while observing their spatial distribution patterns [189]. Furthermore, they have been employed to monitor changes in cell phenotypes occurring during skin organogenesis such as alterations in cluster size, protein localization, and variations in protein expression levels [190, 191]. During embryonic development, the epidermis arises from the surface ectoderm and is distinguished by its expression of KRT8 and KRT18 subsequent to neural formation [190]. Within the basal layer cells of the epidermis, there is an upregulation of markers like KRT5 and KRT14, whereas a downregulation occurs for KRT8 and KRT18 [189]. Upon undergoing terminal differentiation, progenitor cells of cornifying cells migrate toward the suprabasal layer where they exhibit expression of KRT10 [191]. The fibroblasts throughout the dermis exhibit high levels of platelet‐derived growth factor (PDGF) receptor (PDGFR) alpha and PDGFR*β*, which play a crucial role in regulating collagen fiber assembly [192]. Fibroblasts in human skin are primarily characterized by secreted frizzled‐related protein 2 (SFRP2)/dipeptidyl peptidase 4 (DPP4) and flavin‐containing monooxygenase 1 (FMO1)/lymphocyte‐specific protein 1 (LSP1) expression, representing the predominant fibroblast population. The *COL2A1* gene encodes collagen type II alpha 1 chain, predominantly expressed within the connective tissue of the dermis [193]. The hair follicle is situated within the subcutaneous tissue and comprises of the hair bulb, follicular wall, dermal papilla, and medulla. KRT5 and KRT75 are commonly employed as markers for the medulla of the hair follicle, while KRT15 is frequently utilized to label the outer root sheath of the follicle [194, 195, 196, 197]. Additionally, PCNA, cytokeratin (CK) 15, CK5, and KRT17 have demonstrated associations with hair follicle development, and progenitor cell antigen can serve as a marker for hair follicle structure (Table 1) [198, 199].

**TABLE 1:** Related markers for organoids characterization.

Gene	Expression pattern	Applications and characteristics	References
ΔNp63	+	Epidermal basal marker	[162]
AE13	+	Innermost cortex of hair follicles	[163]
COL2A1	+	Connective tissue of the dermis	[193]
COL3A1	+	Dermal marker	[163]
Filaggrin (Flg)	+	Epidermal cornified layers marker; mature epidermal marker	[162, 163, 189]
FMO1/LSP1	+	Fibroblasts	[193]
Involucrin	+	Epidermal granular marker	[162, 189, 191]
Itga6	+	Epidermal marker	[162]
Ki67	+	Hair matrix cell; cell cycle entry marker	[163, 198]
Krt1	+	Epidermal spinous marker	[162, 189, 190]
KRT5	+	Epidermal basal marker; outer root sheath	[162, 163, 189–191, 194]
KRT5	−	Medulla of the hair follicle	[189]
KRT8	−	Epidermis basal cell marker	[189]
KRT8	+	The epidermal marker on the surface of the ectoderm	[189, 191]
KRT10	+	Epidermal spinous marker; progenitor cells of cornifying cells	[162, 189, 190]
KRT14	+	Epidermal basal marker	[162, 189–191, 198]
KRT15	+	Outer root sheath	[194, 198]
KRT17	+	Outer root sheath	[163, 190]
KRT18	+	The epidermal marker on the surface of the ectoderm	[189, 191]
KRT18	−	Basal layer cells of the epidermis	[189]
KRT71	+	Inner root sheath	[163]
KRT75	+	Medulla of the hair follicle	[194]
Loricrin(Lor)	+	Epidermal granular marker; mature epidermal marker	[162, 163, 189, 191]
NFATc1	+	Hair follicles stem cell	[163]
P63	+	Hair matrix cell	[163, 191]
P‐CAD	+	Hair follicle structure	[199]
PCNA	+	Hair follicle development	[198]
PDGFR*α*	+	Dermis fibroblasts	[192]
PDGFR*β*	+	Dermis fibroblasts	[192]
SFRP2/DPP4	+	Fibroblasts	[193]
SOX2	+	Dermal papilla cell	[163]
Sox9	+	Epidermal basal marker	[162]
Vimentin	+	Dermal marker	[163, 191]
*α*‐SMA	+	Dermal sheath	[163]
CDX2	+	Intestinal epithelial cells	[200]
ASCL2	+	Intestinal stem cells	[201]
SCGB3A2	+	Airway epithelial club cells	[202]
FOXJ1	+	Polymorphocytes	[202]
AGER	+	Type I alveolar epithelial cells	[203]
AQP5	+	Type I alveolar epithelial cells	[204]
SFTPB	+	Type II alveolar epithelial cells	[205]

In intestinal organoids, immunohistochemistry and immunofluorescence play significant roles. Caudal type homeobox 2 (CDX2), a transcription factor specific to the intestine, regulates the regeneration and differentiation of intestinal epithelial cells, typically exhibiting expression in the nucleus of adult intestinal epithelial cells [200]. Achaete‐Scute Complex Like 2 (ASCL2) serves as a crucial marker and regulator of intestinal stem cells, being expressed in the stem cells located at the base of intestinal crypts. It plays a vital role in sustaining the stemness and self‐renewal capacity of these cells [206]. Consequently, ASCL2 can also function as an indicator of the intestinal stem cell components within intestinal organoids [201].

Secretoglobin family 3A member 2 (SCGB3A2) is primarily expressed in airway epithelial club cells. In human lung organoids, SCGB3A2 is frequently utilized as a marker to label bronchial organoids that express airway epithelial club cells [202]. The protein encoded by the forkhead box J1 (FOXJ1) functions as a transcription factor primarily involved in the regulatory expression of genes related to cilia formation. In lung organoids, FOXJ1 is frequently utilized as a marker for polymorphocytes [201, 202]. There are two important types of alveolar epithelial cells. Human pulmonary organs possess the ability to differentiate alveoli. Following induction and differentiation, type I alveolar epithelial cells can express the epithelial cell marker, advanced glycosylation end‐product specific receptor (AGER), and aquaporin 5 (AQP5). In contrast, type II alveolar epithelial cells are capable of expressing surfactant protein B (SFTPB) [203, 204, 205].

#### 3.1.4 Radiomics and AI

Radiomics is a rapidly developing field within medical imaging. This technology extracts high‐dimensional quantitative features from medical images using automated algorithms and integrates these features with clinical and biological data to support precision medicine. The essence of radiomics lies in transforming traditional medical images into quantifiable data, thereby uncovering potential information that may be difficult for the human eye to detect [207]. AI, a branch of computer science, focuses on developing technologies that can simulate, extend, and enhance human intelligence. Its core components include machine learning and deep learning [208, 209]. AI combined with radiomics has a wide range of applications in disease research, particularly in the study of tumors. In a study focused on non‐small cell lung cancer, a noninvasive model for predicting immune treatment response was successfully developed through the integration of deep learning and habitat radiomics. This model can effectively distinguish between high‐risk and low‐risk patients, demonstrating significant benefits for clinical applications [210]. Currently, the emergence of organoids has introduced new possibilities for AI and radiomics. In research utilizing PDO endometrial cancer mouse models, magnetic resonance imaging radiomics is employed to capture the early treatment responses of these organ models. This study underscores the significant role of preclinical imaging, including radiological tumor spectra, in assessing the early treatment responses of endometrial cancer models [211]. Research on organoids involves a substantial amount of omics data, including gene expression profiles, proteomics, and metabolomics. AI technologies, such as support vector machines and random forests, can assist in processing these complex datasets and mining potential gene expression patterns. This, in turn, enhances the accuracy of drug screening predictions and offers new insights into understanding cellular development and disease mechanisms [212, 213].

### 3.2 Biomarker Discovery Based on Organoid Analysis

Organoids can be utilized for organoid‐based biomarker discovery, facilitating the identification of novel diagnostic and prognostic markers.

In the study of single‐cell transcriptomics analyzing kidney organoids, a comparative analysis was conducted on two organoid differentiation protocols utilizing scRNA‐seq data, with the aim of devising a new protocol that minimizes the presence of non‐kidney cell types while preserving the integrity of organoid epithelial cells. The study compares single‐cell transcriptomics of 83,130 cells from 65 organoids with that of fetal and adult kidney cells derived from two directed differentiation protocols. Notably, brain‐derived neurotrophic factor and its cognate receptor are expressed in neuronal lineages during organoid differentiation. Inhibiting this pathway resulted in an improvement in organoid formation, reducing the neuronal population by 90% without compromising kidney differentiation. This finding underscores the efficacy of single‐cell technology in characterizing and enhancing organoid differentiation [214].

PDO are frequently employed to identify biomarkers for drug response and precision treatment in cancer patients. Through the establishment of the patient‐derived liver cancer organoid biobank, we identified molecular signatures linked to drug response and predicted potential drug combinations for personalized treatment of patients [215]. In a comparative study of human papillomavirus (HPV)‐associated (HPVA) and non‐HPVA cervical adenocarcinomas (ADC) and squamous cell carcinoma (SCC), the malignancy origins of epithelial cells, angiogenic tip cells, and two subtypes of fibroblasts were examined. Additionally, organoids were utilized to further confirm the premalignant properties of these structural cells. The study also identified diagnostic biomarkers that distinguish ADC from SCC, as well as prognostic biomarkers associated with malignant epithelial cells, tip cells, and the macrophage marker genes *SPP1* and *C1QC* [216].

The integration of precision medicine and biomarkers holds immense promise for revolutionizing healthcare by personalizing treatments based on individual characteristics. Organoids, as a novel tool, offer significant support and expedite the discovery of biomarkers, diagnostic markers, and prognostic markers, thereby enhancing the development of precision medicine.

### 3.3 Organoids Reflect the Molecular Mechanisms and the Intercellular Interactions

#### 3.3.1 Organoids Analysis Signaling Pathways

Organoids can be utilized to investigate signaling pathways and molecular mechanisms involved in tissue development, pathogenesis, and drug toxicity. Understanding these signaling pathways and molecular mechanisms can enhance the accuracy of precision medicine.

Organoids can provide valuable insights into the signaling pathways and molecular mechanisms underlying normal skin development. PDGF is a normal constituent of platelets from which it is released by degranulation of *α* granules at sites of injury [217]. The PDGFb pair stimulated an upregulation of fibronectin and collagen in fibroblasts, leading to an augmentation in the population of vascularized and undifferentiated keratinocytes within the epidermis as well as pericytes [217]. In the process of the cultivation of the skin class organs, PDGF expression level affect skin organoids cell differentiation and structure characteristic and is used to monitor the skin organoids differentiation process table in the cortex and the formation of the dermis [218]. During embryonic skin development and skin injury, PDGF binds to its receptor, PDGFR, activating downstream signaling pathways such as the PI3K/Akt and MAPK pathways. However, in skin organoids, it is necessary to inhibit the PDGF signaling pathway during the early stages of culture. While PDGF promotes the proliferation and migration of endothelial cells, facilitating the formation of new blood vessels, its inhibition in spherical skin organoids is crucial. Although PDGF can maintain the pluripotency and stability of stem cells in skin organoids at this early stage, its presence may adversely affect the vascularization of these organoids. The generation of vascularized skin organoids holds significant implications for clinical applications in regenerative medicine. The signaling pathway of insulin/insulin‐like growth factor (IGF)‐1/PI3K/Akt has been involved in diverse cell activities. In cells, activation of Akt induces cell proliferation and survival, while over activated Akt signaling tends to induce cell transformation [219]. The PI3K/Akt signaling pathway serves as a communication mediator between these two cell populations during hair follicle regeneration and plays a pivotal role in hair follicle formation within skin organoids [220]. In addition, when IGF (such as IGF 2 and IGF 1r) and VEGF (vascular endothelial growth factor) families (such as VEGF 2 and VEGF rs) are inhibited, the size of the skin organoid is significantly reduced [221]. IGF‐1 is essential for embryonic development; its absence can result in developmental defects in skin structures [222]. In skin organoids, while IGF‐1 remains significant, its role shifts to mimicking and maintaining the already established skin structure, rather than contributing to the initial developmental process. Wnt is a key signal pathway in the process of skin organogenesis [223, 224]. In the early stage of skin differentiation, Wnt signaling pathway regulates the early differentiation of epidermal and dermal cell lines [225, 226, 227]. The interaction of epidermal and dermal progenitors, which leads to the activation of the WNT pathway and the formation of pear‐shape structures, has been confirmed in studies of hair follicle organoids [228]. In epidermal 3D organoids culture, functional interfollicular epidermal stem cells with intact stemness and cell connection can be obtained by Wnt3a treatment. In the case of coculture with the supernatant of interfollicular epidermal stem cells, functional organoids with polarity can be generated [229]. The Wnt signaling pathway is critical both during skin development and during the culture of skin organoids. To investigate the role of Wnt in signal pathway of skin, organoids can provide support for the clinical transformation of skin organoids (Table 2).

**TABLE 2:** Signaling pathways influence skin organoid development and differentiation.

Signal pathway	Effect on organoid development and differentiation	References
PDGF signaling	‐ Promotes fibronectin and collagen production in fibroblasts ‐ Augments population of vascularized keratinocytes and pericytes ‐ Necessary for early skin organoid development but requires inhibition for proper vasculature formation	[217, 218]
PI3K/AKT signaling	‐ Mediates communication between epidermal and dermal stem cells during hair follicle regeneration ‐ Crucial for hair follicle formation in skin organoids ‐ Overactivation can lead to cell transformation	[219, 220]
IGF‐1 signaling	‐ Essential for embryonic skin development ‐ Maintains existing skin structure in organoids without influencing initial development ‐ Inhibition of IGF and VEGF reduces organoid size	[221, 222]
Wnt signaling	‐ Regulates early differentiation of epidermal and dermal lineages ‐ Induces formation of skin accessory structures like hair follicles ‐ Critical for maintaining stemness and polarity in epidermal organoids	[228, 229, 230]

Organoids preserve the gene expression patterns that are characteristic of the organs they model. Cerebral organoids have been instrumental in elucidating the molecular mechanisms underlying viral infections such as cytomegalovirus, herpes simplex virus 1, and human immunodeficiency virus 1 [231]. Organoids are utilized to investigate the signaling pathways and molecular mechanisms associated with drug effects. Doxorubicin, a widely used chemotherapeutic agent for various cancers, is known to cause severe side effects in many tissues. The specific mechanisms underlying doxorubicin's cytotoxicity were examined by exposing 3D small intestinal and colon organoids to the drug. In both organoid types, the cell cycle, p53 signaling pathway, and oxidative stress emerged as the most significantly affected pathways [232].

#### 3.3.2 Organoids Analysis the Intercellular Interactions

Organoids serve as excellent in vitro models for investigating cell–cell interactions due to their composition of diverse cell types.

In intestinal organoids, studies involving a large number of intestinal and organoid cells have not only identified additional enteroendocrine cell types but have also defined tuft cells that express inflammatory or neuronal genes, as well as microfold cells that play a role in immune surveillance. Furthermore, interactions among these different cell types contribute to the maintenance of the physiological functions of the intestine [233]. During the process of retinal organoid self‐formation, *VSX* ^2+^ and *PAX6* ^high^ cells exhibited distinct marker expression within the monolayer, demonstrating a patterned distribution among the cells. Concurrently, it was observed that cell–cell adhesion plays a critical role in both cell survival and the establishment of tissue structure, a phenomenon potentially regulated by rock‐mediated actomyosin dynamics [234].

Organoids serve as powerful tools for investigating intercellular interactions by recapitulating the complexity of in vivo tissues. Their capacity to model these interactions provides unparalleled opportunities to advance our understanding of biological processes, disease mechanisms, and therapeutic strategies. As organoid technology continues to evolve, the incorporation of a broader range of cell types and enhancements in structural fidelity will further improve their physiological relevance and applicability.

The technical and theoretical foundations of organoid‐guided precision medicine are closely intertwined. The integrated application of bioinformatics and big data, including high‐throughput screening and genomic analysis, provides essential tools for the collection and analysis of data on organoids. Biomarker discovery and validation are critical for ensuring the accuracy and reliability of these models. Concurrently, theoretical foundations rooted in cell–cell interactions, molecular mechanisms, and precision medicine offer a robust framework for translating these models into clinical applications.

## 4 Clinical Transformation in Precision Medicine: From organoid‐Based Disease Modeling to Personalized Therapy

**FIGURE 5:**
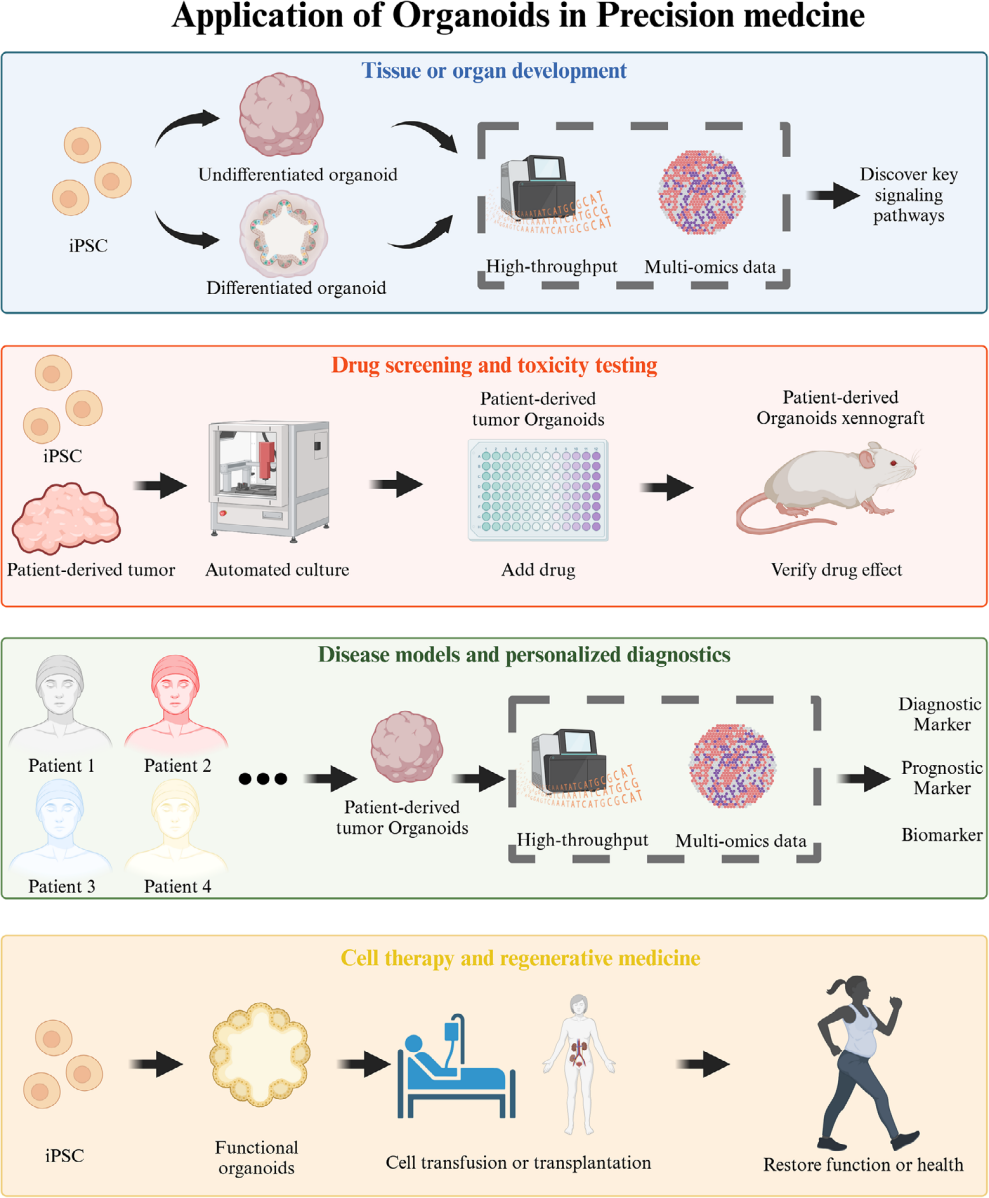
The application of organoids in precision medicine. Organoids are utilized in various fields, including precise medical applications in tissue and organ development models, drug screening and toxicology testing, disease modeling, personalized diagnostics, and cell therapy and regenerative medicine. The black arrows represent the sequence of processes. Image created in BioRender.com.

Organoids are utilized in models of tissue or organ development, drug screening and toxicity testing, disease modeling, and personalized diagnostics, as well as in cell therapy and regenerative medicine. They offer significant promise for advancing disease research and enhancing personalized diagnosis (Figure 5).

### 4.1 Tissue or Organ Development Model

Due to their high tissue specificity and physiological relevance, the application of organoids in models of tissue or organ development is rapidly expanding, positioning them as important tools in various areas of biomedical research.

Given the ethical and regulatory challenges associated with human embryo research, in vitro skin organoids present significant potential as valuable resources for investigating skin development. Skin organoids can be employed to explore specific signaling pathways that govern the differentiation of ectodermal and mesenchymal cell lines into mature skin organs [190]. Moreover, the differentiation and development of skin organoids closely resemble that of embryonic skin, enabling exploration of the impact of microenvironment and cell‐to‐cell interactions on stem or progenitor cells during skin development, including complex processes induced by skin appendages [19]. An additional advantage offered by employing skin organoids is the circumvention of species differences introduced by animal models, thereby reducing reliance on animal experimentation [19, 143]. Testicular organoids have been utilized to study testicular physiology and development, shedding light on the regulation and function of the spermatogonial stem cell niche, germ cell proliferation, and differentiation [235]. In recent years, cardiac organoids have significantly contributed to the study of cardiac development models. Currently, self‐organizing, heart‐like cells have been established from human PSCs. These cells exhibit specific and stereotyped characteristics, evolving into chamber‐like structures that contain cavities. This advancement provides a solid anatomical basis for modeling both cardiac development and cardiac disease [236].

### 4.2 Drug Screening and Toxicity Testing

Organoids have emerged as a valuable tool in drug screening and toxicity testing, offering a more ethical and efficient alternative to traditional animal testing methods.

In recent years, the development of in vitro drug screening technologies utilizing PDOs has garnered significant attention. In the context of gastric cancer, biobanks of patient‐derived gastric cancer organoids have been established through organoid technology, which preserves the structural and functional characteristics of the original tumors. These gastric cancer organoids exhibit varying responses to different chemotherapy agents. RNA sequencing revealed that tumor suppressor genes and associated pathways were upregulated in gastric cancer organoids that were sensitive to 5‐fluorouracil (FU) or oxaliplatin, whereas genes and pathways related to proliferation and invasion were elevated in those exhibiting chemotherapy resistance. The drug response results obtained from PDO were validated in PDO xenograft mice and aligned with actual clinical responses in 91.7% of gastric cancer patients [237]. In human CRC tumor‐derived organoids, a combination of a repurposed drug library and computational drug prediction was employed to test 335 drugs, resulting in the identification of 34 drugs with anti‐CRC effects [238]. In addition, a human liver organoid‐based screening model for analyzing drug‐induced liver injury pathology at the organoid resolution has been established. This system has successfully developed an organoid‐based assay with multiplexed readouts that measure viability, cholestatic toxicity, and/or mitochondrial toxicity, demonstrating high predictive values. The assay was applied to 238 marketed drugs at four different concentrations, yielding a sensitivity of 88.7% and specificity of 88.9% [239].

In conclusion, organoids represent a revolutionary tool for drug screening and toxicity testing, offering a more physiologically relevant model for evaluating therapeutic efficacy and safety. Their application could facilitate more informed and personalized treatment strategies, improve drug development processes, and enhance our overall understanding of human biology. As research in this field progresses, organoids are anticipated to assume an increasingly significant role in pharmacology and toxicology.

### 4.3 Disease Models and Personalized Diagnostics

Organoids can effectively recapitulate essential features of disease biology, making them ideal platforms for investigating the molecular and cellular underpinnings of various diseases. Their applications span a wide array of conditions, including infectious diseases, genetic disorders, cancer, and degenerative diseases. Skin organoids can simulate the cellular composition and physiological structure of human skin, and provide a more realistic microenvironment to study the occurrence and development of these diseases, such as microbial infections [240]. A study conducted cocultivation experiments of skin organoids with SARS‐CoV‐2 to investigate its infectivity toward the skin [241]. Skin organoids can also be used for Localized scleroderma and other skin diseases research. The human‐induced PSC‐derived epithelial and mesenchymal organoids model in a 3D culture system, can significantly reduce the degree of skin fibrosis. In particular, organoids enhance the activity of epidermal stem cells in the localized scleroderma skin and promotes the regeneration of sweat glands and blood vessels [242]. Organoid technology has emerged as a powerful tool for modeling corneal disease, allowing for the study of organ development and disease pathophysiology in a 3D system [243]. Corneal epithelial organoids recapitulate the cellular and gene expression characteristics of human corneal epithelium. An in vitro dry eye model can be established by inducing hyperosmotic stress on these organoids. Notably, dry eye‐related inflammatory factors are significantly expressed in corneal epithelial organoids exhibiting dry eye disease, suggesting that this model effectively reflects the characteristics of functional dry eye disease [244].

In personalized diagnostics, organoids play an essential role in predicting individual patient responses to drugs and tailoring customized treatments. This is particularly important in cancer and rare genetic diseases. Patients with biliary tract cancer (BTC) show different responses to chemotherapy, and there is no effective way to predict chemotherapeutic response. A study involving 61 PDOs from 82 tumor patients with BTC demonstrated that these organoids exhibited histological and genetic characteristics closely resembling those of the corresponding primary BTC tissues. Furthermore, the BTC‐PDOs displayed varied responses to different chemotherapy regimens, including gemcitabine, cisplatin, 5‐FU, and oxaliplatin. The outcomes of the PDO drug screening were corroborated by PDO‐based xenograft experiments, revealing that 92.3% (12 out of 13) of BTC patients exhibited actual clinical responses [245]. Moreover, gene expression signatures derived from BTC‐PDOs were identified and correlated with various drug responses, leading to the establishment of gene expression panels aimed at predicting chemotherapy responses in BTC patients [237]. Cystic fibrosis, a disease caused by mutations in the cystic fibrosis transmembrane conductance regulator (*CFTR*) gene, which encodes the cystic fibrosis transmembrane conductance regulator, shows promising insights through recent studies. Forskolin‐induced swelling of in vitro‐expanded human control and cystic fibrosis organoids quantitatively correlates with forskolin‐induced anion currents observed in freshly excised ex vivo rectal biopsies. The function of the CFTR F508del mutant protein can be restored through incubation at low temperatures, as well as by the application of *CFTR*‐restoring compounds. This relatively straightforward and robust assay will enhance diagnosis, facilitate functional studies, and support drug development and personalized medicine approaches in the context of cystic fibrosis [246].

Overall, organoids present significant potential for personalized diagnostics and medicine, providing a platform for tailored treatments and disease modeling that reflects individual patient characteristics. The application of organoids across various medical fields is continually expanding, thereby facilitating personalized approaches in healthcare.

### 4.4 Cell Therapy and Regenerative Medicine

Organoids have emerged as promising tools in cell therapy and regenerative medicine. Their capacity to closely replicate the structural and functional characteristics of human tissues and organs positions them as an ideal platform for advancing therapies designed to repair or replace damaged tissues.

Organoids provide a novel strategy to deliver functional cellular or tissue components to repair damaged tissues or restore lost functions. This is particularly useful in conditions where natural regeneration is limited or absent. Organoids can generate specific cell types relevant for transplantation and therapeutic purposes. These cells can help repair damaged organs or restore functionality.

Producing functional *β* cells in vitro has generally proven to be a challenge. Current research indicates that islet‐like organoids derived from in vitro cultures exhibit glucose responsiveness and insulin secretion. When transplanted into diabetic mice, these organoids have been shown to reverse the disease. Consequently, pancreatic organoids containing insulin‐producing beta cells hold promise for transplantation in diabetic patients, potentially restoring insulin production and regulating blood glucose levels [247]. Immune cell therapy involves the use of immune cells, such as T cells or natural killer cells, to treat diseases Currently, human thymus organoids derived from iPSC have been successfully generated, demonstrating the capability to support the de novo generation of a diverse population of functional human T cells. T cells from iPSC‐thymus‐engrafted humanized mice exhibit the ability to mediate both cellular and humoral immune responses, including the initiation of robust proinflammatory responses upon T cell receptor engagement, the inhibition of allogeneic tumor graft growth, and the facilitation of efficient immunoglobulin class switching [248]. Thymic organoids have the potential to produce effective T cells and are anticipated to improve the immune function of patients with immunodeficiency diseases through cell therapy.

Organoids can be used as a regenerative medicine platform to repair or replace damaged tissues or organs. Skin damage resulting from surgery, trauma, or burns can have profound physical and psychological implications. Human iPSC‐derived skin organoids offer a potential cellular source for skin grafts, presenting a promising avenue for the treatment of challenging wound healing and permanent loss of skin appendages [19]. Recent advancements propose the utilization of human iPSC‐derived skin organoids that seamlessly integrate with mouse skin to facilitate facial repair and reconstructive procedures [40]. Moreover, by combining 3D printing techniques with skin organoids, it is feasible to generate layered structures using these organoids [249]. Furthermore, considering the variations in facial tissue composition and hair coverage across different regions of the face [250], personalized human iPSC‐derived skin organoids with or without hair follicles can be employed to match the characteristics of the original site [67]. Currently, options for skin organoid transplantation in the treatment of burns and wound healing are limited. One potential method is single‐cell suspension grafting. This process involves obtaining a single‐cell suspension derived from a skin organoid, which is then transplanted into a silicone grafting chamber placed on the muscle fascia within the wound. The chambers are removed 7 days posttransplantation [40]. For skin organoids generated using 24‐well membrane inserts, creating a single‐cell suspension is not required. Instead, the harvested skin organoids can be directly utilized as grafts for organoid transplantation, which may also expedite the process of skin healing [251]. An alternative viable solution for skin organoid transplantation involves the integration of skin organoids with 3D printing technology to create 3D bioprinting skin organoids. Subsequently, these 3D bioprinting skin organoids were directly transplanted into skin defects located on the backs of mice, resulting in effective repair of full‐thickness skin defects [252]. The application of these skin organoids in tissue engineering and reconstruction holds significant importance in achieving aesthetic restoration, functional rehabilitation, scar reduction, and minimizing immune rejection within facial skincare. It provides an invaluable tool within the field of regenerative medicine.

Cholangiocyte organoids can repair bile ducts after transplantation in the human liver. Cholangiocyte organoids present a promising approach for repairing bile ducts following transplantation in the human liver. These organoids exhibit plasticity and can re‐establish their in vivo characteristics upon transplantation into the biliary tree. Transplantation experiments utilizing cholangiocyte organoids in an in vitro constant‐temperature perfusion model of human liver cell implantation have demonstrated their potential to repair human intrahepatic ducts after transplantation [253]. In addition, the human airway organoid exhibits the ability to rapidly condense and self‐organize, forming discrete epithelial and endothelial structures that demonstrate mechanical strength and stability during long‐term culture. This capability represents a significant step toward lung regeneration [254].

Organoids possess significant potential in cell therapy and regenerative medicine, particularly for the repair or replacement of damaged tissues and the generation of functional cells for transplantation. Recent advancements in organoid bioengineering, bioprinting, and gene‐editing technologies suggest that organoid‐based therapies are poised to revolutionize personalized medicine and regenerative therapy in the near future.

### 4.5 Preclinical Animal Experiments and Clinical Trials

Organoids have long been regarded as a promising alternative to preclinical animal experiments and clinical trials [255]. The use of organoid‐based 3D in vitro systems has already yielded initial results in this regard (Table 3). For example, skin organoids can be employed to study UV‐induced photodamage on hair follicles. Preclinical animal experiments were conducted to evaluate the effectiveness of exosomes isolated from human umbilical cord blood‐derived mesenchymal stem cells in mitigating hair follicle damage in skin organoids [163]. Neonatal hypoxic injury can be treated with minocycline, an US FDA‐approved small molecule. In brain organoids subjected to hypoxic conditions, minocycline has been shown to mitigate the adverse effects of hypoxia on dorsal brain genes, oligodendrocytes, and neuronal progenitors [256]. However, reports on the application of organoids in preclinical animal experiments and clinical trials remain scarce. For adrenal organoids, large animal models have been employed to assess the feasibility of adrenocortical cell transplantation (ACT). Autologous porcine ACT involved performing bilateral adrenalectomy, followed by cell isolation, culture, and the subsequent intracutaneous injection into a skin location that was prepped with a biodegradable temporizing matrix foam. The transplanted adrenocortical cells demonstrated an ability to survive and multiply within the intracutaneous area, as well as a capacity to self‐organize into distinct tissue organoids that exhibited characteristics of typical adrenal histological architecture [257].

**TABLE 3:** Clinical trials and cases of organoids.

Organoids types	Diseases	Application	Trial registration	References
Tumor‐derived organoids	Colorectal cancer	Drug screening	NCT03251612	PMID: 37143108
Pancreatic organoid	Pancreatic ductal adenocarcinoma	Pathway verification	NCT03454035	PMID: 36346366
Patient‐derived organoids	Nonmuscle invasive bladder cancer	Drug screening	NCT05024734	PMID: 37170307
Patient‐derived organoids	Colorectal cancer	Drug screening	NCT03668431	PMID: 36702949
Patient‐derived organoids	Non‐small cell lung cancer	Pathway verification	NCT02609776	PMID: 32414908
Patient‐derived organoids and xenografts	Non‐small‐cell lung cancer	Drug screening	NCT02535507	PMID: 30596880
Patient‐derived organoids	Metastatic colorectal cancer	Drug screening	NCT01607957	PMID: 36864254
AEC2‐fibroblast coculture organoids	Interstitial lung disease	Pathway verification	NCT05195918	PMID: 38980870
Intestinal organoids	Cirrhosis and acute‐on‐chronic liver failure	Pathway verification	NCT03202498	PMID: 38621924
Patient‐derived organoids	Neuroendocrine carcinoma	Drug screening	NCT02695459	PMID: 33776923
Human ileal organoids	Human ileum	Pathway verification	ISRCTN11327221	PMID: 38896603
Patient‐derived organoids	Advanced gastro‐esophageal adenocarcinoma	Drug screening	NCT00824785	PMID: 33199443
Intestinal ex vivo organoid	Celiac disease	Pathway verification	NCT03256266	PMID: 32103108
Patient‐derived organoids	Neuroendocrine prostate cancer	Drug screening	NCT01799278	PMID: 30232224
Patient‐derived organoids	Lynch syndrome	Drug screening	NCT02052908	PMID: 32641470

## 5 Clinical Challenges: Translational Bottlenecks and Ethical Considerations

Organoid research has emerged as a groundbreaking tool in the study of human diseases and developmental biology. However, despite their potential, researchers continue to encounter several limitations, including ethical issues, heterogeneity, and challenges related to standardization.

### 5.1 Ethical Issues

Research on organoids, particularly those related to humans, still faces several ethical issues that require resolution. First, the use of human ESCs in constructing organoids raises significant ethical and legal disputes, as the procurement of ESCs necessitates the destruction of embryos. Although adult normal specimens and iPSCs are now utilized in organoid research instead of ESCs to circumvent the ethical disputes associated with embryo destruction, ethical concerns regarding privacy protection persist [242, 258]. Research involving organoids, including the generation of PDOs from tumor tissues, may entail the personal information and biological samples of research subjects [259]. Ensuring the security of this information and protecting the privacy of samples from potential leaks is an urgent ethical issue that requires resolution. Research on organoids may give rise to various social and ethical issues, including ethical dilemmas surrounding organ transplantation and the redefinition of human identity and dignity. These concerns can lead to public apprehension and disputes, potentially hindering the progress of research. For instance, current studies utilizing organoid technology to create mini‐brains exhibit developmental characteristics akin to those of a fetus several months old. Although these organoids are smaller in size and differ significantly in structure and function, the presence of neural connections and electrical activity in brain organoids raises ethical questions about whether they have developed any form of perception [260].

### 5.2 Heterogeneity

Organoids, as 3D in vitro models, are capable of simulating the structure and function of organs. However, heterogeneity presents a significant challenge in organoid research. One source of this heterogeneity is the starting cells used to derive organoids, which are typically obtained from iPSCs or ASCs. Factors such as the epigenetic state of these stem cells, the accumulation of gene mutations, and variations among individual donors can all contribute to this heterogeneity [261]. In PDTO, individual donors can contribute to intratumoral heterogeneity, which in turn affects the drug resistance of tumors. Therefore, it is essential to consider the heterogeneity of organoids when utilizing them for disease modeling, drug screening, and cell therapy [262]. Although organoid research has brought new hope for disease treatment and drug development, concerns regarding heterogeneity persist, particularly regarding the long‐term safety and potential risks associated with organoids. These risks include the possibility of triggering an immune response and the potential for tumor formation. Consequently, the application of organoids in cell therapy and regenerative medicine presents significant challenges [261, 263].

### 5.3 Standardization

Organoids exhibit significant potential in biomedical research, drug development, and personalized medicine. However, the standardization of organoids still faces numerous challenges. In addition to genetic variability arising from different donor cells, subtle changes in culture conditions, such as the specific conditions for organoid culture and mechanical stimulation, can also considerably impact the morphology and functionality of organoids [264, 265]. Furthermore, technical variations among operators, including factors such as cell quantity and differentiation induction, can lead to batch‐to‐batch discrepancies in organoid production [266]. These variations pose challenges in meeting the high‐throughput requirements necessary for effective drug screening. Consequently, there is a pressing need for equipment capable of large‐scale and automated production to satisfy the demands for high‐throughput generation of standardized organoids [267, 268]. Additionally, the current preparation and research of organoids lack a unified standard and quality control system—encompassing parameters like size, organoid structure, and biomarker expression levels—resulting in inconsistencies in organoid standards across different laboratories [265]. Overall, organoids present significant potential in disease modeling, regenerative medicine, and cancer therapy, but standardization challenges in culture methods and production remain key areas of focus for advancing research and clinical applications in the field.

## 6 Conclusion and Prospects

Disease research and drug development have traditionally depended on in vitro cell cultures and animal models [269, 270, 271]. However, the use of homogenized cell lines and animal models often results in a uniform biological background, which can lead to unsatisfactory outcomes when translating scientific research findings to individual patients [272]. This limitation represents a significant challenge in the field of precision medicine. Organoids have emerged as a crucial tool in precision medicine. These organoid cultures, derived from both healthy and pathological tissues, including cancerous tissues, offer numerous advantages in drug discovery and personalized medicine [273, 274]. In the realm of cancer biology, tumor organoids derived from patient tissues can be cultured in vitro for extended periods while preserving the genetic diversity and mutational characteristics of the original tumors [275, 276, 277]. Consequently, by culturing tumor organoids from patient‐specific samples, researchers can directly support precision medicine strategies, including personalized drug screening and treatment efficacy assessment. PDTOs have become highly accurate models for studying cancer and for the development of new precision medicine approaches. Similar to tumor organoids, normal tissue organoids developed using stem cells and organ tissue technology play a crucial role in the field of precision medicine. In the context of skin organoids, these structures have been precisely engineered. The resulting skin organoids serve as a valuable platform for studying skin development processes [278]. Skin organoids can serve as personalized research models for skin diseases [240]. Specifically, skin organoids derived from human PSCs provide a pathophysiological model for studying SARS‐CoV‐2 infection. They not only offer insights into the relationship between COVID‐19 and hair loss but also demonstrate the utility of skin organoids in investigating viral infections and screening potential therapeutics [241]. IPSC‐derived skin organoids have been employed to model systemic scleroderma and to examine the antifibrotic effects of selective estrogen receptor modulators, further illustrating the value of skin organoids in modeling complex diseases and assessing therapeutic candidates [279]. Similarly, various other types of organs are developed and utilized for related medical research, including liver, cardiac, and lung organoids, among others [154, 157, 280]. By studying organoid disease models in patient‐relevant contexts, there is hope to expedite the translation of therapies from the laboratory to clinical application. Consequently, organoids represent an innovative tool for advancing precision medicine from the laboratory to the clinic.

The future of organoid technology is driven by continuous innovation, including the development of high‐throughput organoid generation systems, automated culture platforms, and advanced bioprinting technologies. These advancements aim to create more physiologically relevant tissue models in a scalable and reproducible manner [267, 268]. Such technological progress will not only enhance the efficiency and accessibility of organoid‐based workflows but also facilitate the creation of more complex systems, such as multiorgan‐on‐a‐chip models, to study interorgan interactions and systemic drug responses [280]. In the realm of personalized medicine, organoid systems hold immense promise for developing patient‐specific treatment strategies. By integrating multiomics data—encompassing genomics, transcriptomics, proteomics, and metabolomics—organoids can unveil molecular insights into disease progression and identify actionable therapeutic targets [281, 282]. PDOs, when combined with genome editing tools like CRISPR and high‐content drug screening, can guide tailored drug combinations and dosages, thereby reducing the risk of therapy failure and minimizing unwanted side effects [111]. Furthermore, organoid‐guided approaches can be instrumental in addressing chemotherapy resistance, rare diseases, and immune modulation by enabling researchers to simulate patient‐specific responses in vitro [283, 284]. Looking ahead, the convergence of organoid technologies, AI, and multimodal data analytics is poised to revolutionize the design of personalized drug regimens. This integration will enable not only highly precise, organoid‐guided individualized treatments but also enhance our understanding of complex biological systems. Collectively, these advancements have the potential to bridge the gap between bench and bedside, accelerating translational medicine and reimagining the future of healthcare.

Despite the significant potential of organoids in precision medicine, these models currently face substantial challenges. As a result, organoid models remain largely in the laboratory stage, with limited preclinical animal studies and clinical trials reported. First, because organoids are cultured under artificially controlled conditions, such as specific culture scaffolds and exogenous cytokines, it is challenging to maintain the balance between proliferation and differentiation of various cell types within the organoids. Furthermore, different cell types require distinct cellular microenvironments. Although it is feasible to employ culture protocols utilizing various media at different time points, it remains difficult for a single culture medium to satisfy the needs of all cell types [285]. Second, while it is currently possible to generate organoids that resemble normal tissues and exhibit certain functions, high‐fidelity replication of tissue structure and function in vivo is still lacking. Although this discrepancy does not impede in vitro research, it significantly hinders the clinical application of organoids for in vivo transplantation. Additionally, the limited lifespan of organoid cultures, which can only be maintained for a short duration, represents a major bottleneck in organoid research [286]. For instance, skin organoids reach the end of their culture life after 150 days [19]. This phenomenon is primarily attributed to the absence of blood vessels in the cultured organoids. Consequently, the acquisition of materials and metabolic waste removal for the cells within the organoids depend solely on the exchange of culture fluid, leading to cell death due to nutritional deficiencies. Finally, generating organoids from human PSCs is a time‐consuming and challenging process [285]. This complexity hinders the widespread production of large quantities of standardized organoid models, thereby complicating the transition of organoid applications from the laboratory to clinical settings.

To address the limitations of organoids and enhance their application in precision and regenerative medicine, collaborative efforts from various experts will be essential. In the field of materials science, the exploration of more suitable biomaterials that can accurately simulate the in vivo state of cells and provide a more appropriate microenvironment may facilitate the efficient generation of organoids. Recent advancements in the development of vascularized organoids have been noteworthy, with researchers investigating various strategies to construct functional blood vessels within these 3D tissue models [287, 288, 289]. By integrating blood vessel structures with typical human cortical cell types, researchers successfully created a functional brain organoid model that sustained viability for over 200 days [287, 290]. Although these vascularized organoid models do not yet fully replicate real vascular structures and their culture lifespan remains limited, this work demonstrates that the incorporation of vascular structures can enhance nutrient supply and waste removal. This suggests that vascularized organoids may offer a viable approach to making organoid models, including skin organoids, more suitable for clinical applications [291]. Consequently, future research should focus on continuously exploring innovative techniques and identifying more appropriate biological materials to deepen our understanding of organoid development, accurately simulate the body's microenvironment, and strive to overcome existing challenges to expedite the transition of organoids from the laboratory to clinical settings.

In summary, the organoid model serves as an accurate and reliable in vitro system that effectively elucidates the developmental processes of tissues and the potential underlying mechanisms of disease occurrence within the laboratory environment. Additionally, it holds promise as a potential transplant option to facilitate personalized treatments in regenerative medicine. Despite the significant potential of organoid models and the growing number of related laboratory studies, the journey toward clinical translation remains a considerable challenge. We hope that this review will contribute to the advancement of precision medicine from the laboratory to clinical practice, guided by organoid models.

## Author Contributions

*Conceptualization*: Boqiang Tao, Weiwei Liu, and DongxuWang. *Investigation*: Boqiang Tao and Weiwei Liu. *Project administration*: Weiwei Liu and Dongxu Wang. *Resources*: Weiwei Liu. *Supervision*: Weiwei Liu and Dongxu Wang. *Visualization*: Boqiang Tao. *Writing original draft*: Boqiang Tao. *Writing review and editing*: Boqiang Tao, Xiaolan Li, Ming Hao, Tian Tian, Yuyang Li1, Xiang Li, Chun Yang, Qirong Li, Qiang Feng, Hengzong Zhou, Yicheng Zhao, Weiwei Liu, and Dongxu Wang. All authors have read and approved the final manuscript.

## Ethics Statement

The authors have nothing to report.

## Conflicts of Interest

The authors declare no conflicts of interest.

## Data Availability

The data that support the findings of this study are available in the article.
